# Image registration method using representative feature detection and iterative coherent spatial mapping for infrared medical images with flat regions

**DOI:** 10.1038/s41598-022-11379-2

**Published:** 2022-05-13

**Authors:** Hao-Jen Wang, Chia-Yen Lee, Jhih-Hao Lai, Yeun-Chung Chang, Chung-Ming Chen

**Affiliations:** 1 Department of Biomedical Engineering, National Taiwan University, Taipei, Taiwan; 2 Department of Electrical Engineering, National United University, Taipei, Taiwan; 3 Department of Medical Imaging, National Taiwan University Hospital and National Taiwan University College of Medicine, Taipei, Taiwan

**Keywords:** Breast cancer, Image processing, Machine learning

## Abstract

In the registration of medical images, nonrigid registration targets, images with large displacement caused by different postures of the human body, and frequent variations in image intensity due to physiological phenomena are substantial problems that make medical images less suitable for intensity-based image registration modes. These problems also greatly increase the difficulty and complexity of feature detection and matching for feature-based image registration modes. This research introduces an automatic image registration algorithm for infrared medical images that offers the following benefits: effective detection of feature points in flat regions (cold patterns) that appear due to changes in the human body’s thermal patterns, improved mismatch removal through coherent spatial mapping for improved feature point matching, and large-displacement optical flow for optimal transformation. This method was compared with various classical gold standard image registration methods to evaluate its performance. The models were compared for the three key steps of the registration process—feature detection, feature point matching, and image transformation—and the results are presented visually and quantitatively. The results demonstrate that the proposed method outperforms existing methods in all tasks, including in terms of the features detected, uniformity of feature points, matching accuracy, and control point sparsity, and achieves optimal image transformation. The performance of the proposed method with four common image types was also evaluated, and the results verify that the proposed method has a high degree of stability and can effectively register medical images under a variety of conditions.

## Introduction

Medical image registration is a major area of image registration research, as medical images are often multitemporal and have strict image registration requirements. Among the different types of medical imaging, infrared imaging is particularly useful because of its substantial advantages, namely that it is low cost, does not involve the use of radiation, is painless, and can provide information solely using energy emitted from the human body. Because multitemporal infrared images can reflect long-term changes in the body’s heat map, they are considered a potentially powerful tool for chemotherapy evaluation and early cancer detection^1,2^.

However, infrared images pose many challenges in image registration. These images only reflect the surface temperature of the human body, have low contrast, have insufficient intensity resolution, and provide heat map information that can vary substantially among individuals. In such images, multiple flat areas with uniform gray levels may appear. Defining effective feature points in these areas is difficult. In addition, there are few related studies in infrared breast image registration, so it is still important and worthy research topics^3,4^.

Image registration is a critical step in computer vision, pattern recognition, and medical diagnosis. Image registration methods can be divided into two categories, namely intensity-based and feature-based methods^5,6^. Intensity-based registration methods are often used for small-displacement registration tasks, such as any two consecutive frames of a video and are effective for non-rigid image registration. Intensity-based registration methods are often used for small-displacement registration tasks, such as any two continuous frames, in a video and are effective for non-rigid image registration^7,8^. However, if intensity-based registration is used for images with highly variable image intensity such as grayscale images, accurate registration is difficult. Feature-based registration has been extensively researched and applied^5,6,9^. Because it could easily achieve the registration tasks as large displacements, brightness changes, or rotation changes by good selection and optimization of feature detection and matching. In other words, for feature-based registration, whether the method used in the process is appropriate is critical. Feature-based registration includes three steps. First, feature points detection: representative features from the source image and the target image are detected. Second, feature matching: small windows at feature points in the source image are compared with windows of the same size at feature points in the target image. Matched feature are control points used for the image transformation. Finally, transformation: the control point are used to determine a transformation model which registers the image, that is, registers the source image to the target image. Each step is closely connected and inseparable.

### Feature point detection

In previous studies, classical feature point detection methods include corner-point and spot methods (local extreme points)^5,6,10^. However, corner detection methods (e.g., FAST, SUSAN, MSFD and curvature scale-space (CSS)^11–13^) can generate rich and accurate corners, but are susceptible to inaccuracies for images with homogenous backgrounds and are therefore unsuitable for low-contrast images. Spot detection methods such as Hessian’s determinant or maximum stable extreme region^14–16^ and the feature detection algorithms SIFT^17,18^ and SURF^19^ rely on extreme points in a region to detect feature points. Aldana-Iuit et al. introduced a feature detector named Sadder^20^. The detector can effectively detect saddle conditions. Compared with traditional methods, the Sadder detector can obtains higher repeatability^21^. But the algorithm of this detector depends on intensity profile of image. Thus, these algorithms are likely to overdetect or underdetect feature points in low-contrast regions of grayscale infrared medical images. Therefore, slight changes in the thermal mode can cause spot detection failure or inaccuracy. In recent years, deep learning has also been used to detect feature points, which can be roughly divided into supervised learning^22–24^ and unsupervised learning^25–27^. However, the use of deep learning inevitably requires a lot of data as training. Not applicable to this study.

In 2017, Lee et al.^28^ developed an algorithm to solve the problem of image registration for infrared images of humans. However, the algorithm cannot detect the features of flat areas, leading to an uneven distribution of feature points and a deviation in registration for images with many flat areas. Even though feature point detection in flat areas is critical for the registration of human images, no research has been conducted on algorithms with this capability. Therefore, the present study developed a feature point detection algorithm that can effectively detect features in flat areas of infrared images and ensure the uniform placement of feature points in each position of the image for more accurate image registration.

### Feature matching

Because of changes in human physiology or the environment and the characteristics of infrared images, feature matching is more difficult in infrared images than in in visible light images. To overcome these problems, this study used the relationships among feature points for matching and mismatch removal. In similar studies Zhou et al.^29^ utilized a factorized graph-matching method that avoids the calculation of the pairwise adjacency matrix, and they decomposed the adjacency matrix into submatrices to reduce the computational burden. However, Zhou’s method is more suitable for rigid images. In many matching techniques, descriptors are created for similarity measurement which improve the effectiveness of feature point matching. In the research published by Gesto-Diaz et al.^30^, different classic descriptors, such as SURF, MI, and HOG, were evaluated for the potential for use in matching. The pattern of local gravitational force local descriptor is handcrafted float-type descriptors prosed by Bhattacharjee et al. can be regarded as a combination of force magnitude and angle and shown promising performance^31^. Wang et al.^32^ based on scene geometric structure constraints and numerical statistical features of feature invariant scale transformation proposes a method aimed at improving the performance of feature point matching stage named GeoMatch. It can greatly improve the matching speed of SIFT descriptors.

However, although the above method can achieve matching, it does not consider how to perform Mismatch removal in the case of matching errors. Mismatch removal is also a critical step in improving the registration quality. Fischler et al*.*^33^ introduced the RANdom SAmple Consensus (RANSAC), which iteratively estimates the parameters of the mathematical model from a data set. Thresholds are used to determine whether the data are outliers, and outlying i.e., mismatched) points are removed. However, image registration of breast infrared images is a nonrigid transformation, but the threshold approach in the RANSAC implementation is only applicable to rigid transformations. In 2012, Ma et al.^34^ published a method of mismatch removal through coherent spatial mapping (MR-CSM), which they developed using thin plate splines (TPSs)^35^. Their algorithm uses a gentle deformation constraint model during the iterative process and removes matching pairs that are far away from each other. Same authors have realized a follow-up technique named VFC (Robust Point Matching via Vector Field Consensus) using the similar idea as CSM. In the article published in 2014^36^, it claimed to have a high matching accuracy rate.

Since these two (MR-CSM and VFC) methods are similar concepts, considering that the matching time will account for most of the overall alignment time in this research, when the total time complexity of the VFC algorithm is high, VFC is not used as the basis Development method. Because MR-CSM is applicable to image pair cases that contain nonrigid transformations and outperforms many state-of-the-art methods, it was used in the present study to develop a novel feature point matching strategy.

### Transformation model

Transformation is the last step of image registration. The transform function estimates the displacement vector of each pixel. This vector corresponds to mapping that is used to transform the source image. The process may or may not consider matching pairs. However, matching pairs can be an important reference for image transformation. Reddy et al.^37^ devised a rigid transformation method based on the Fourier–Mellin transform. Matching pairs are not considered in the transformation; spectrum phase correlations are used to calculate the position of the largest peak value of the inverse fast Fourier transform of the phase difference to obtain the transformation parameters, such as the displacement, rotation angle, and magnification. As an example of nonrigid transformation, Chui et al.^38^ introduced the TPS robust point matching (TPS-RPM), which uses TPSs and matches points according to point distance. However, during the matching process, all points must be matched without consideration of whether a point is an outlier, which results in the risk of a poor final transformation.

Diffusion models are other useful nonrigid transformation methods that do not consider matching pairs. Thirion^39^ introduced the Demon method. The boundary of the target image is regarded as the registration target, and the boundary of the source image is regarded as a deformable grid. The source image is further diffused and deformed by the optical flow method. The method consists of two iterative steps: First, the differences in pixel values and the gradient are used to calculate the displacement of each pixel. Second, a Gaussian filter is used to smooth the displacements between the pixels. Vercauteren et al.^40^ developed diffeomorphic Demons, in which Lie group theory is used to modify the update rule of the Demons displacement so that the update is faster and the transformation is less distorted. However, such algorithms only consider the differences in grayscale values. Hence, they are intolerant to changes in grayscale.

In 2011, Brox et al.^41^ presented the large-displacement optical flow (LDOF). The method first involves the use of the segmentation method developed by Arbelaez et al.^42^, which is based on the watershed algorithm, to divide the target and source images into subregions, with the center of each subregion regarded as a feature point. Next, matching is conducted with a SIFT descriptor. The obtained matching pairs. Control points, are then substituted into the LDOF energy function to calculate the vector value of each pixel. This algorithm can realize large displacements to obtain pixel-level image mappings.

The main contributions of the present study include feature point detection in flat areas and feature matching optimization. The feature point detection algorithm improves the registration model’s capability of obtaining sufficient feature points with a uniform distribution. The new matching strategy uses MR-CSM and matching point addition strategies combined with LDOF and uses two-stage iteration to correct matching errors and determine optimized matching pairs. These optimized matched pairs are used as control points of the transformation. The optimization process in this method achieves superior image registration.

- Develop a feature point detection algorithm for flat regions of infrared images.
- Combine MR-CSM with LDOF to develop a matching strategy and optimal transformation estimation method for an infrared image registration algorithm.
- E valuate the proposed algorithm’s performance and applicability in comparison with those of other developed methods.

## Results and discussion

**Figure 1:**
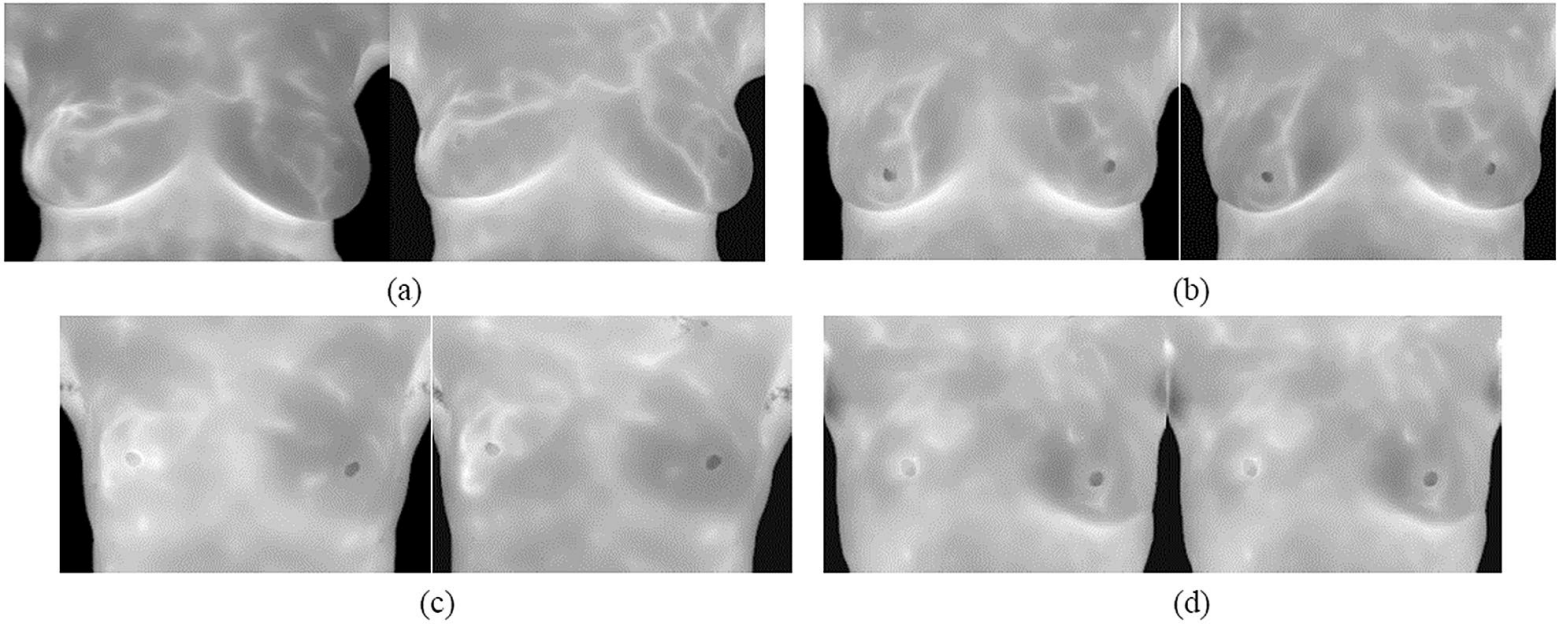
Results of four registration situations: (**a**) TYPE I, (**b**) TYPE II, (**c**) TYPE III, and (**d**) TYPE IV.

To evaluate the performance of the proposed registration method on breast infrared images, the reliability and stability of the proposed method was demonstrated under four registration situations in three key steps (feature point detection, feature matching, and transformation). The four situations were large displacement with many flat regions (TYPE I), small displacement with few flat regions (TYPE II), small displacement with many flat regions (TYPE III), and large displacement with few flat regions (TYPE IV), shown as Fig. 1. The proposed method’s feature point detection performance was compared with that of the image registration algorithm developed by Lee et al. and the SIFT and SURF algorithms. The transformation results were compared with those of Lee et al. image registration algorithm and an optical flow algorithm to ensure the validity and reliability of the proposed method.

### Feature point detection

To characterize the effectiveness of the proposed method’s detection of feature points, the proposed method was compared with the classic feature point detection algorithms SIFT and SURF. Table 1 shows the detected feature points (red dots on the infrared image) under the four registration situations.

**Table 1:**
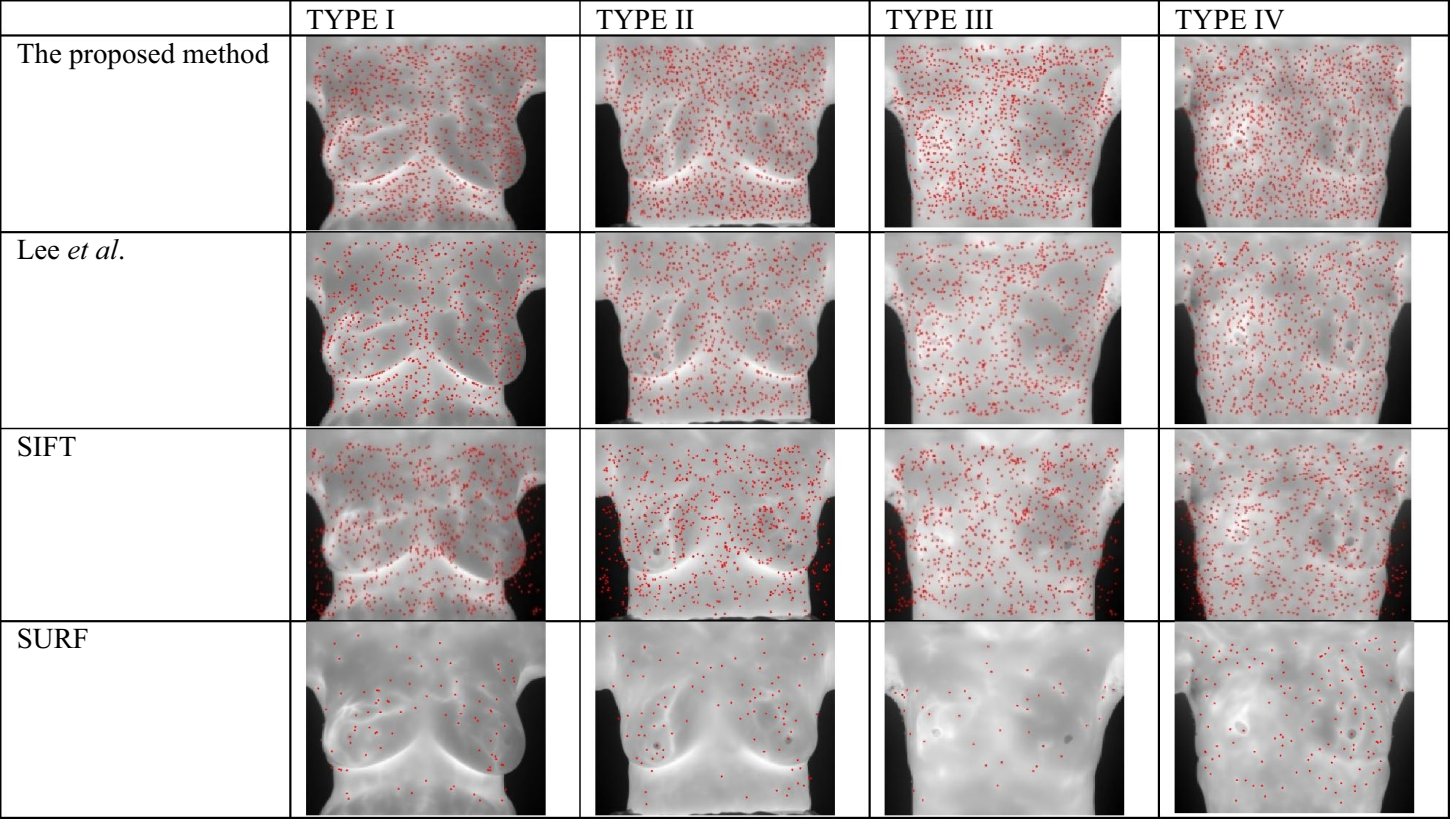
Detected feature points under the four registration conditions.

Table 1 shows that SURF detected the fewest feature points, whereas the performance of the other three methods varied according to the feature detection area in image. The number of feature points detected with the method developed by Lee et al. was similar to that of SIFT, but the method developed by Lee et al. could not detect feature points in flat areas. The SIFT method resulted in the excessive clustering of feature points in some areas and could not detect feature points in flat areas. The visualization results demonstrated that regardless of the type of alignment, the method proposed in the present study can detect numerous evenly distributed feature points, which is beneficial in the subsequent registration steps.

**Figure 2:**
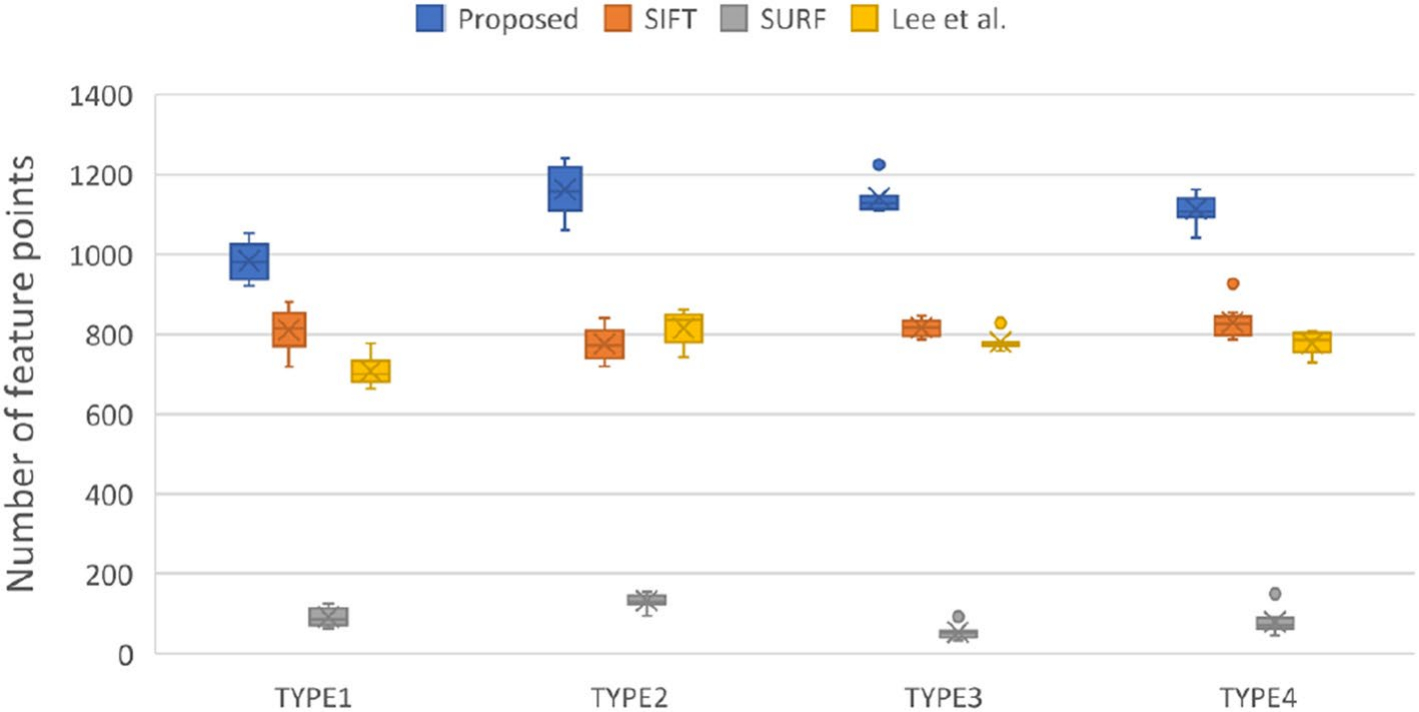
Compare the number of feature point detection by four methods in each types.

**Figure 3:**
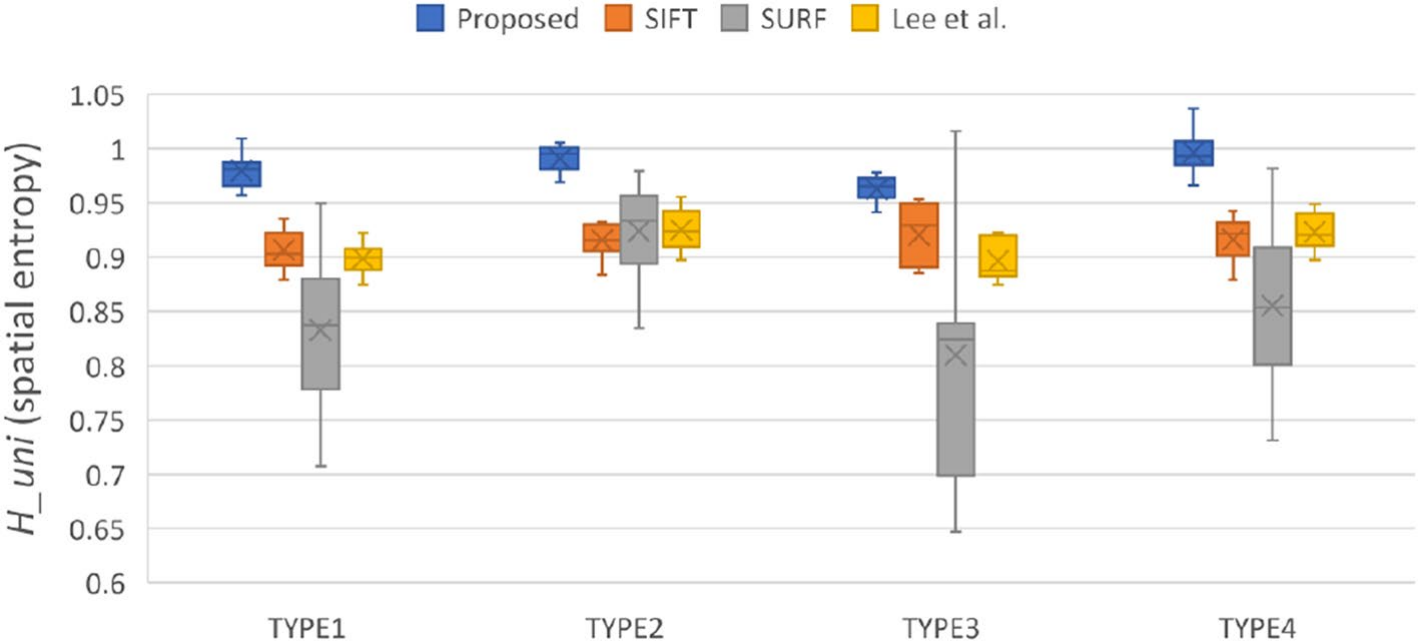
Compare the feature point distribution uniformity calculated by four methods in each type.

**Figure 4:**
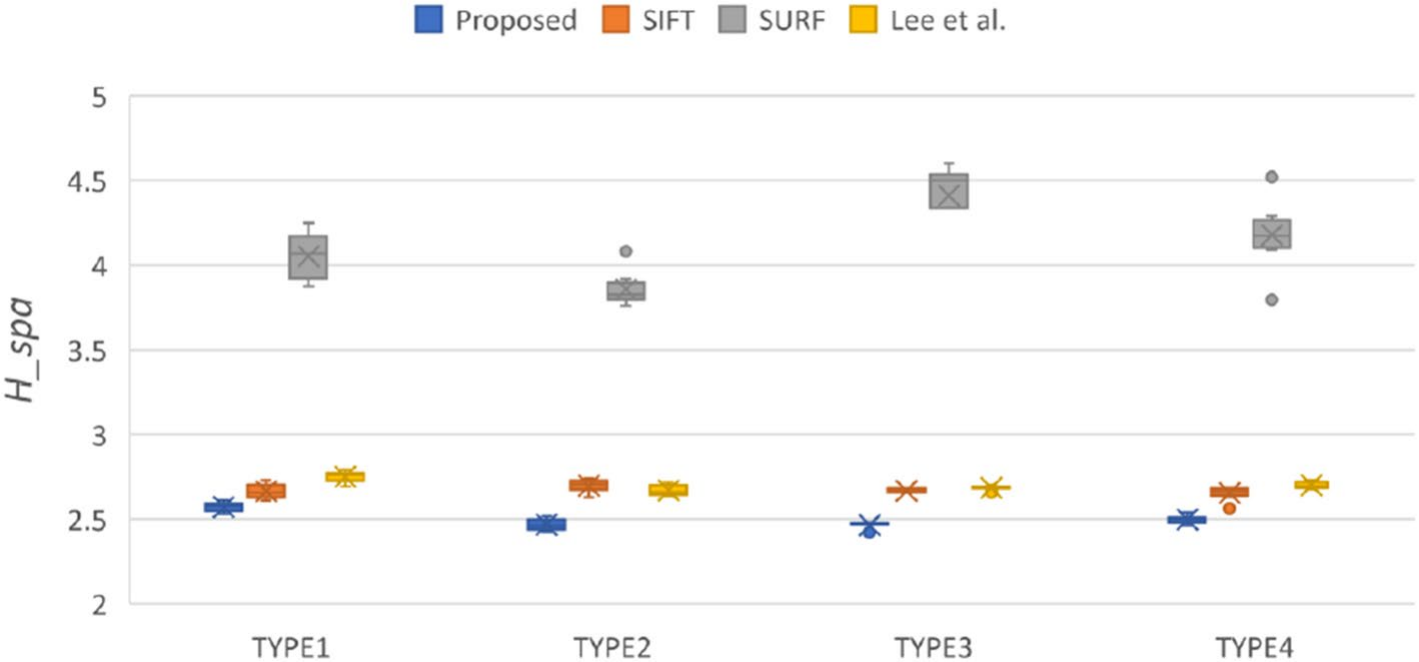
Compare the sparseness of detected feature points calculated by four methods in each type.

Figures 2, 3, and 4 display the quantitative results of feature point detection. Figure 2 shows the number of detected feature points and offers a visualization of the results in Table 1.

The SURF method detected the fewest feature points, whereas the method developed by Lee et al. and the SIFT method detected a similar number of points. In all registration situations, the proposed method detected the largest number of feature points. In particular, for TYPE I (the most difficult registration task), the proposed method exhibited the best feature point detection performance.

This study adopted the definition of spatial entropy proposed by Martínez et al.^43^ to demonstrate that higher feature point distribution uniformity leads to a better image transformation. The spatial entropy is ${H}_{uni}=\frac{\langle \mathrm{r}\rangle }{{r}_{ran}}$, which measures how far the real distribution of pairs is from a completely random one. The spatial entropy is a descriptor of the uniformity of feature point distribution the mean nearest neighbor distance is $\langle \mathrm{r}\rangle =\frac{1}{\mathrm{n}}\sum_{i=1}^{n}{\mathrm{dis}\_\mathrm{X}}_{i}$, where ${\mathrm{dis}\_\mathrm{X}}_{i}$ is the distance between a feature point *i* and its nearest neighbor. The variable *n* is the number of feature points. If ${I}_{pixels}$ represents all pixels of Image *I*, then the expected average distance between nearest neighbors is given by ${r}_{ran}=\frac{1}{2}\sqrt{\frac{{I}_{pixels}}{n}}$. Figure 3 shows that the method proposed in this study determines the set of feature points with the highest uniformity of the four methods compared. The SURF method does not lag behind other methods significantly in terms of uniformity, and some outliers of box plot even perform similarly to the proposed method.

The definition of spatial entropy was modified to obtain a parameter that could be used to evaluate the sparsity of feature points, ${H}_{spa}=\frac{\langle \mathrm{r}\rangle }{{A}_{per}}$, where ${A}_{per}=\frac{\mathrm{n}}{{I}_{pixels}}$ is the number of feature points per pixel. Figure 4 shows that the proposed method yielded the least sparse feature points among the methods evaluated.

For all four registration tasks, the proposed method detected a larger number of feature points than did the other methods.

The obtainment of a dense feature distribution that is effective for image registration depends on the detection quantity, distribution uniformity, and overall density of features, which are displayed in Figs. 2, 3, and 4, respectively. Although Fig. 3 shows that SURF has high uniformity, Figs. 2 and 3 demonstrate that this result was attributable to insufficient detection quantity, leading to an improved uniformity score when calculated on the basis of the expected average point distance. However, a model cannot be considered to have good feature point detection when only one of the conditions is met, but rather when all conditions are met. The SIFT method’s performance was the most similar to that of the method developed by Lee et al*.*, but the two methods had some differences, as indicated in Table 2. The SIFT method detects extreme points by using the Gaussian difference of images; however, this strategy causes feature point clustering and therefore hinders subsequent feature point matching. Although SIFT could detect feature points in some flat areas, the number of feature points detected in many flat areas remained sparse. Because the algorithm used by Lee et al. relies on detecting corners and intersections in the thermal map, the detected features primarily surround heat pattern and cold pattern (flat regions); therefore, the feature points detected within these regions are sparse. By contrast, the proposed method detected feature points in these flat regions and improve the image registration performance that would otherwise be poor due to the lack of control point pairs in these regions. The proposed method met all three of the feature point detection conditions. In particular, even for the TYPE I and TYPE III images, which had many flat regions, the proposed method yielded satisfactory feature point detection results.

**Table 2:**
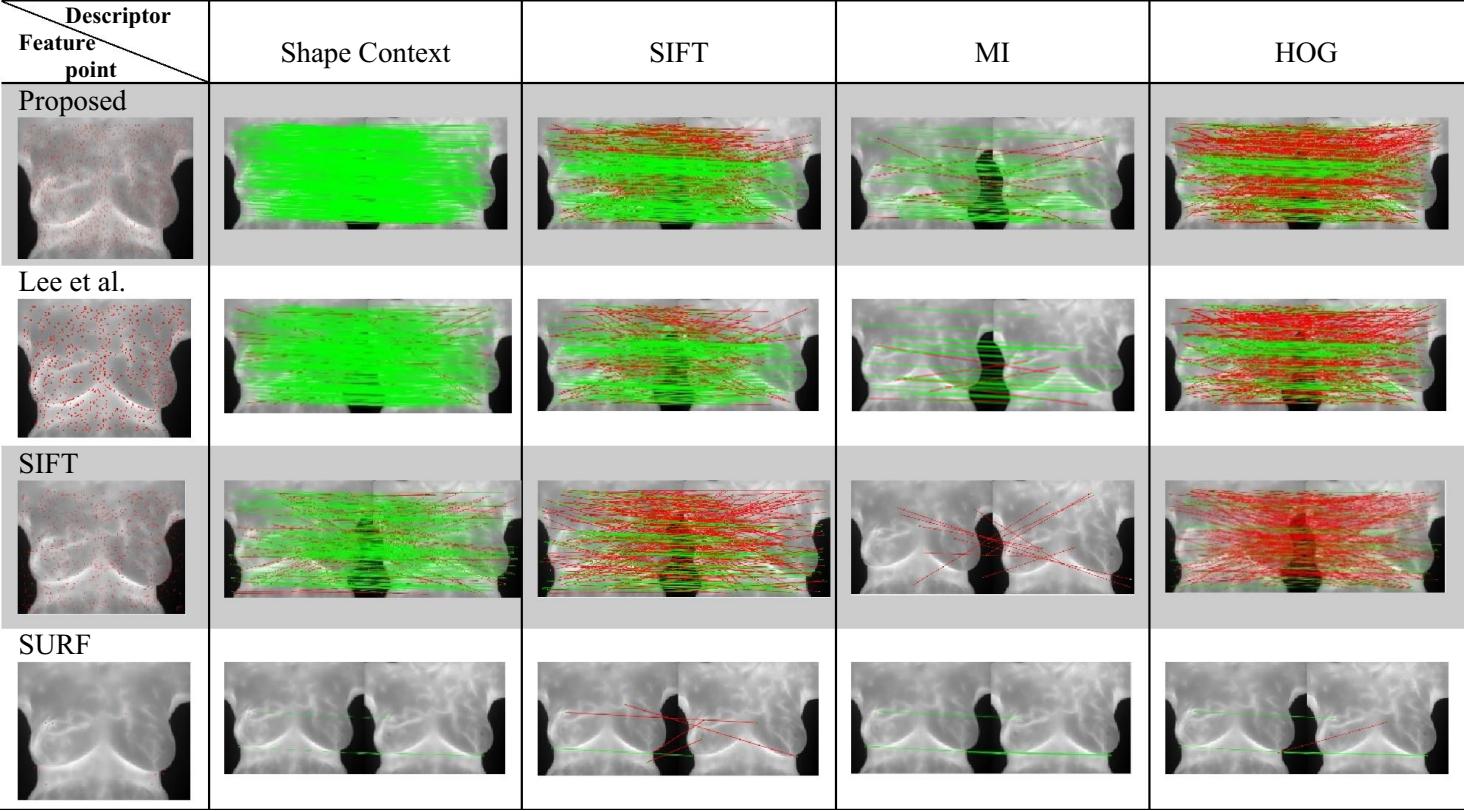
Visualize the comparison of matching result through different combinations of feature points and descriptors.

### Feature point matching

The proposed feature point detection algorithm performed feature matching by using each of four classic feature descriptors, and was comparable to the method developed by Lee et al., SIFT, and SURF with the same descriptors. Table 2 presents the feature matching results. The proposed feature point detection algorithm with the Shape Context (SC)^44^ descriptor correctly matched the most points correctly. The proposed algorithm performed well by detecting additional feature points in the flat regions to overcome a lack of control point pairs in flat regions, therefore achieving the dense feature distribution required for high image registration performance.

**Figure 5:**
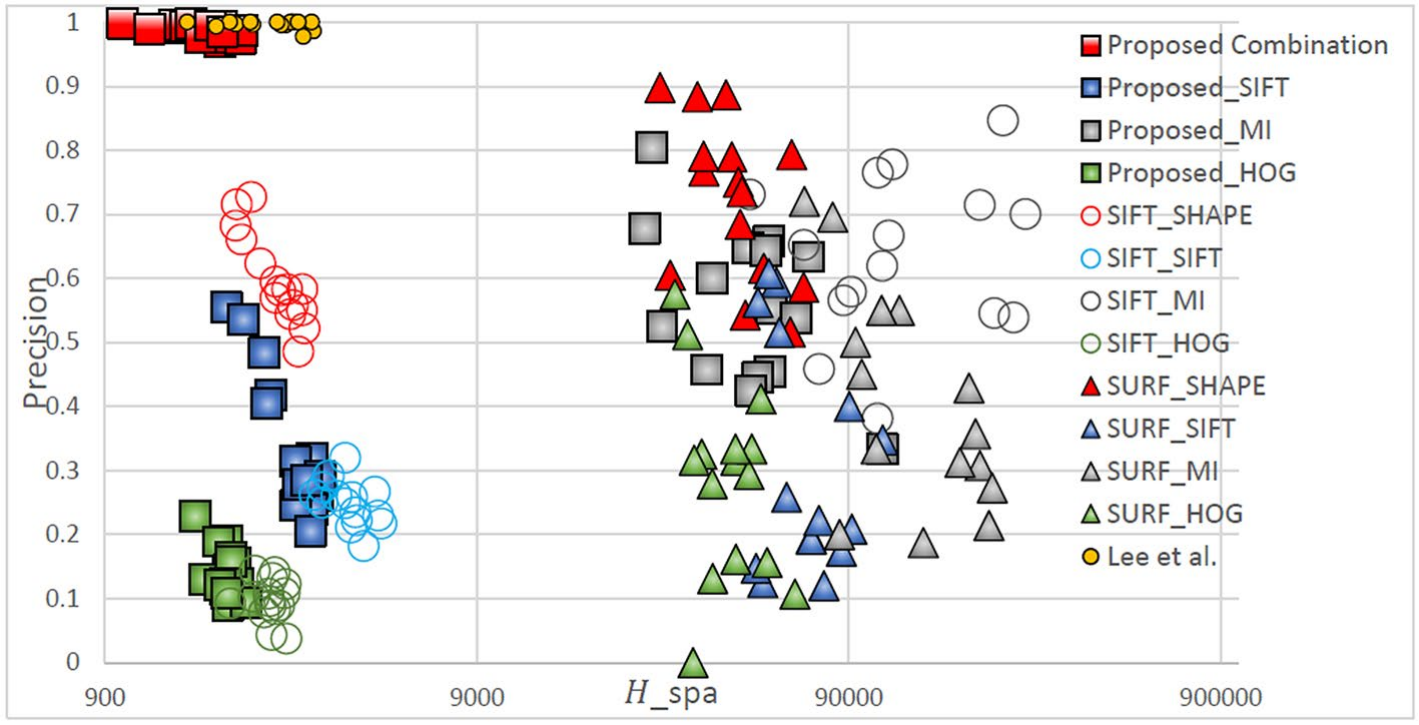
Quantitative comparison of matching in TYPE I through different matching combinations.

**Figure 6:**
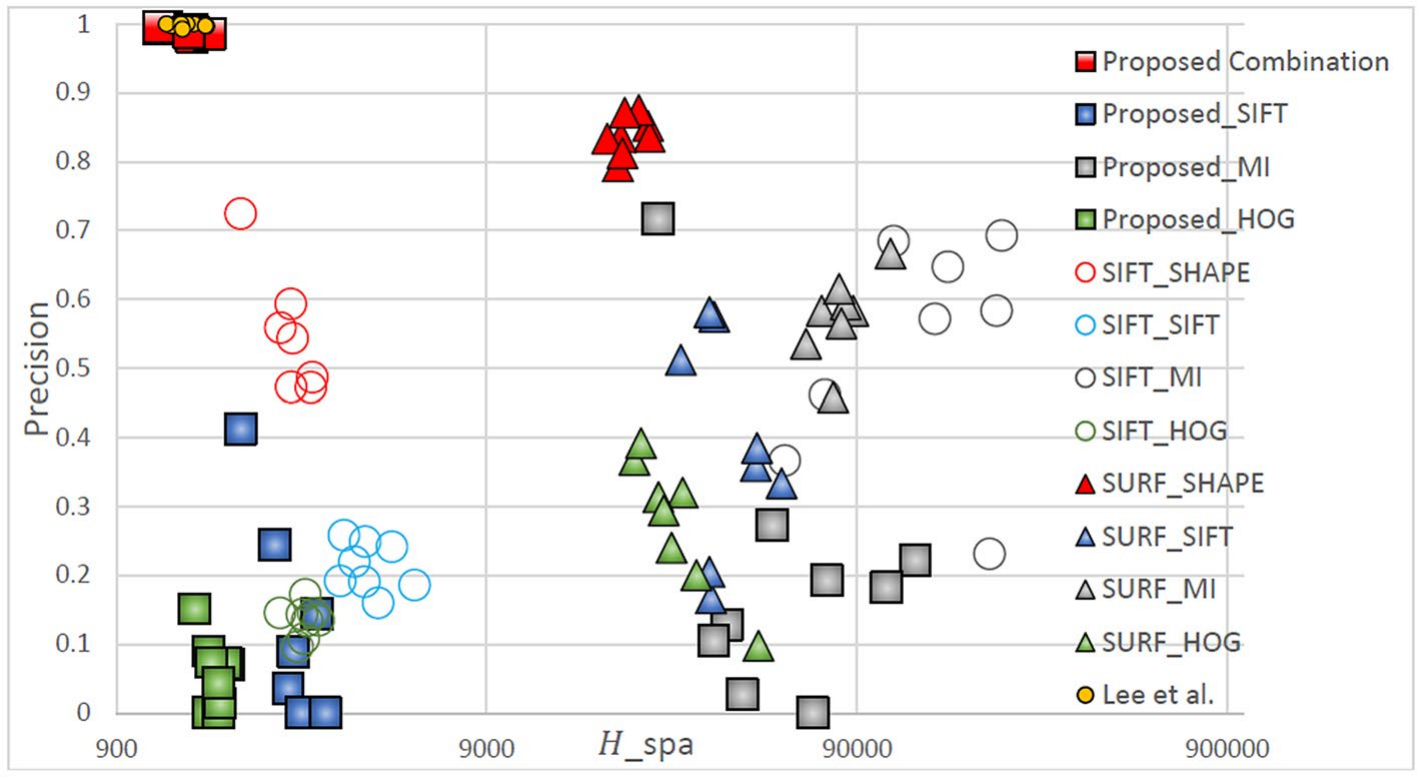
Quantitative comparison of matching in TYPE II through different matching combinations.

**Figure 7:**
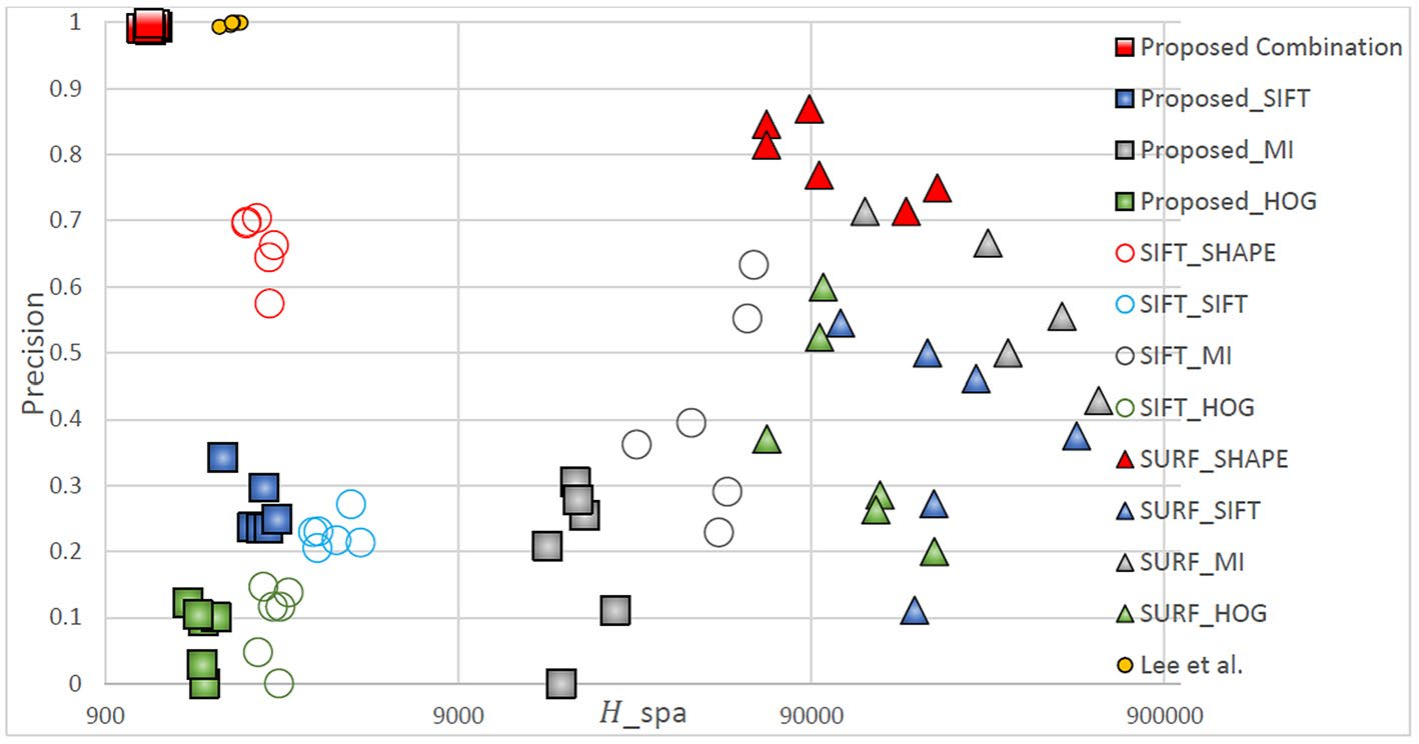
Quantitative comparison of matching in TYPE III through different matching combinations.

**Figure 8:**
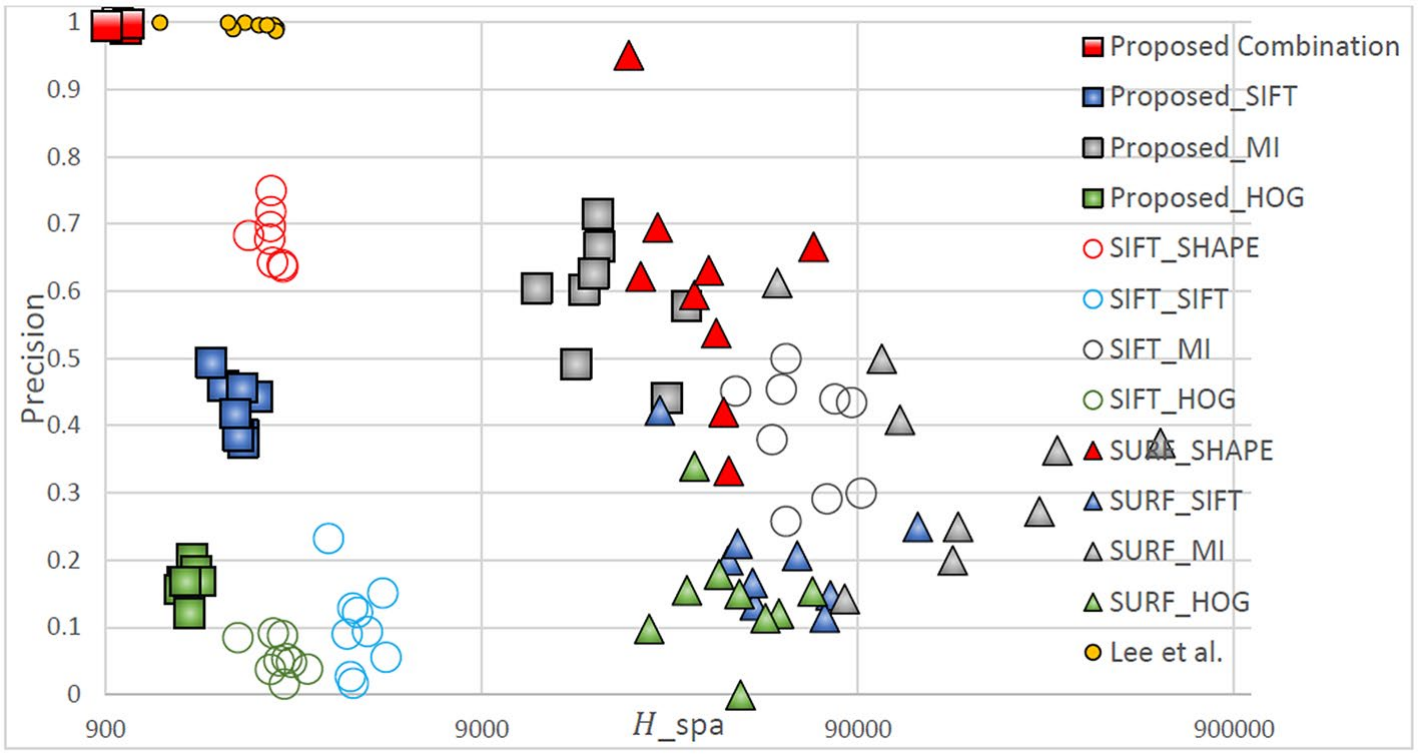
Quantitative comparison of matching in TYPE IV through different matching combinations.

Table 2. Green lines represent correctly matched point pairs, whereas red lines represent incorrectly matched point pairs. The number and accuracy of points matched by the proposed method were both highest across all feature descriptors. For all feature detection algorithms, the matching performance was highest for the Shape Context descriptor, followed by SIFT, HOG, and MI. For all feature descriptors, the matching performance was highest when the proposed method was used, followed by when the method developed by Lee et al*.*, SIFT, and SURF were used. Figures 5, 6, 7, 8 display quantitative data concerning the feature matching results for different registration situations. In each figure, different colors and shapes represent different combinations of feature point detectors and descriptors. The red boxes indicate the proposed method using the Shape Context descriptor. The yellow dots indicate the method developed by Lee et al. using the Shape Context descriptor. The vertical axis of the chart represents the matching accuracy (higher is better), and the horizontal axis represents the sparsity of the matching points *H*spa (lower is better). The points in the top left corner of the chart represent the performance of an optimal feature point–descriptor combination. Precision and recall have often been used in research to evaluate matching performance. However, as recall is the ratio of correct matches to all matching pairs, methods such as SURF have a misleadingly high recall value due to insufficientn feature detection. Therefore, in Fig. 5 through Fig. 8, precision and sparsity (Hspa) are used instead to perform a two-dimensional evaluation of the matching performance. When the precision value is larger, the Hspa value is smaller, which means better matching performance.

Figure 5 displays the matching performance of different feature point-descriptor combinations in the TYPE I (large displacement and many flat regions) registration task. It can be clearly seen from the figure that the evaluated results of the proposed method are concentrated in the upper left corner of the graph, indicating that it has both low sparsity and high matching accuracy, and thus has the best matching performance. The method developed by Lee et al*.* achieved equivalent precision but higher sparsity compared with those achieved by the proposed method due to a lack of features detected in flat regions. This disparity was particularly noticeable in TYPE I images with more flat regions.

The figure shows the performance of matching sparsity *H*_*spa*_. Because the proposed method, the method of Lee et al., and SIFT detect more feature points compared with SURF, they have lower matching sparsity when combined with the Shape Context, SIFT, and HOG descriptors, but not with the MI descriptor since it relies on measuring the consistency of gray-scale statistics in the two image regions. In TYPE I, the large displacements cause the grayscale distribution of the medical infrared image to be more likely to change, and the fact that there are many flat regions is not conducive to uniqueness for a descriptor based on grayscale statistics. Therefore, matching sparsity is higher.

By contrast, each feature point detection method had relatively good matching precision with Shape Context (red marks) and the MI descriptor (gray marks). Shape Context had better similarity measurement capabilities than the MI descriptors for the same matching sparsity. SIFT descriptors measure the similarity of 128-dimensional gradient information, and HOG descriptors measure 16-dimensional gradient information. The gradient information on infrared medical images changes easily in TYPE I images, and thus is difficult to achieve correct matching.

Figure 6 displays the matching performance of different feature point-descriptor combinations in the TYPE II (small displacement and fewer flat regions) registration task. Because TYPE II images were the least difficult images to register all methods exhibited relatively low sparsity (*H*_spa_). However, the detection of numerous feature points reduces the matching accuracy if a feature descriptor with insufficient similarity measurement capability is used. Because the method developed by Lee et al. can detect enough feature points, its matching performance is similar to the proposed method. Comparing Fig. 6 with Figs. 5, 15, and 16, the overall matching sparsity data is observed to be relatively low for TYPE II images. However, when the matching sparsity is low and the number of detected feature points is large (i.e., for the proposed method, Lee et al., and SIFT) if the matching descriptors have insufficient similarity measurement capabilities, the matching accuracy rate will be relatively low By contrast, choosing a rich and discriminative descriptor such as the Shape Context descriptor increases the matching accuracy.

Figure 7 displays the matching performance of different feature point-descriptor combinations in the TYPE III (small displacement and less flat regions) registration task. The method of this research still has the best matching performance. A comparison of the matching performance for TYPE III images with that for TYPE II demonstrates that the matching sparsity is significantly reduced. The fundamental reason is that the method of Lee et al. cannot detect enough feature points in images with more flat regions and therefore the matching sparsity performance has decreased. For medical infrared images with more flat areas, descriptors that rely on metric grayscale statistics and gradient information, such as MI, SIFT, and HOG have difficulty establishing unique of the descriptors. Therefore, fewer feature points are accurately matched and the matching sparsity performance is reduced. The matching accuracy is affected by the similarity measurement ability of the descriptor and the matching sparsity. Therefore, similarly to TYPE I and TYPE II images, the selection of a good feature point–descriptor combination is necessary for superior matching performance.

Figure 8 displays the matching performance of different feature point–descriptor combinations the TYPE IV (small displacement and less flat regions) registration task. Similar to the previous three registration tasks, the matching combination proposed in this study has the best matching performance. In TYPE IV images with fewer flat regions, although the method of Lee et al. detects almost as many feature points as the method of this research, the performance is still worse. Therefore, the proposed method is most capable of registration of images with large displacements.

Matching performance was evaluated both visually and quantitatively, and the proposed method was found to generate higher-quality feature point matching pairs as image control points for image registration. Table 2 shows how visual observation can be used to evaluate matching accuracy, and the proposed method clearly matched many feature points correctly. The proposed method exhibited the highest matching performance of all the algorithms for each descriptor. Table 2 also indicates that the quality of feature point detection affected the matching performance and therefore affected the registration performance. Table 2 also presents the registration performance of image matching when a good feature point–descriptor combination is selected and the matching method is properly set.

### Transformation

Table 3 indicates the effects of different transformation methods on the registration results. The upper left cell of the table shows the source image and target image. The left side of the pictures in the remaining cells displays a comparison of the registration results from the target image and the source image through the use of canny edges. Green lines represent the overlaid part of the edge, white lines represent the edge of the source image, and red lines represent the edge of the target image after transformation with a particular method. On the right side of each cell, the transformed target image is displayed. The table compares optical flow transformations that do not rely on feature points, displays the results of registration with different feature points for LDOF and TPS methods that rely on feature point transformation, and uses MI to illustrate the quantified registration performance.

**Table 3:**
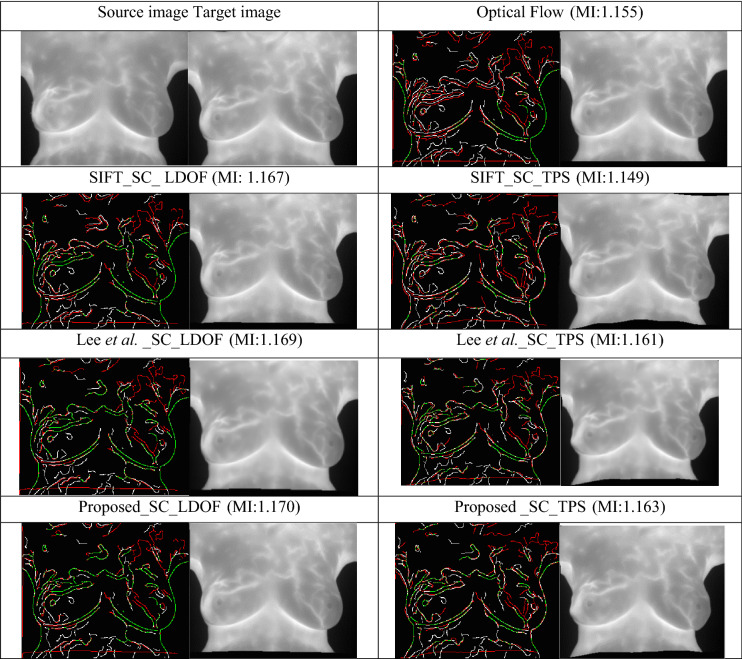
Comparison of registration results through various methods.

p.s. The words separated by underscores represent the method of “Feature points”、 “descriptor”、and “transformation ” in order that used for registration; MI = mutual information; SC = Shape Context; TPS = thin plate spline.

Table 3 reveals that the best registration results were obtained when good feature point detection and matching mechanisms were selected and the best transformation model was used. During image transformation, TPS relies heavily on the detection of feature points. Therefore, registration results vary considerably according to the number of feature points. Because OF methods do not rely on feature points, they have disadvantages in the registration of images with distortion. Results from LDOF demonstrate that a combination of feature base (feature point matching) and intensity base can achieve large displacements.

Canny edges were also used in this study to evaluate the image transformation performance of each method for each registration task. The results are displayed in Figs. 17, 18, 19, 20. A higher proportion of green lines in an image represents a closer match, and therefore higher registration performance.

Table 4 shows the registration performance measured by MI evaluation of the different methods in the four registration types, and calculates the significance statement to show whether there is a difference in performance between the different methods. Table 5 shows the time performers of the proposed method in the four registration types. The results show that each case can be registered within 40 s, outperforming the method of Lee et al. Although the method of this study is inferior to the method of optical flow in terms of time efficiency, the method of this study can achieve more accurate alignment results, which is more in line with the purpose of this study.

**Figure 9:**
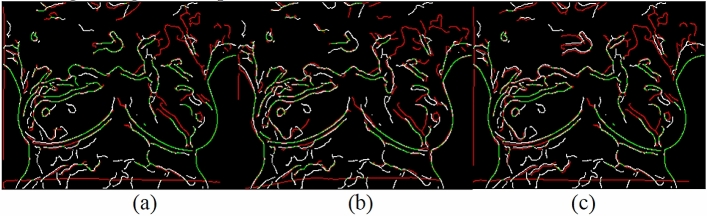
Canny overlays of TYPE I images obtained with (**a**) the proposed (**b**) Lee et al*.* (**c**) Optical flow.

**Figure 10:**
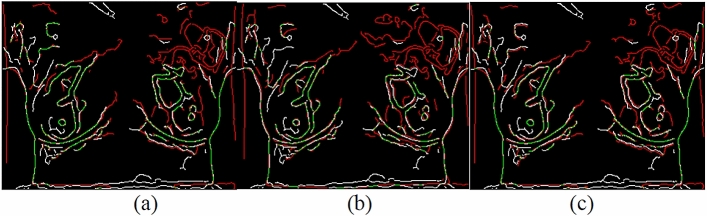
Canny overlays of TYPE II images obtained with (**a**) the proposed (**b**) Lee et al*.* (**c**) Optical flow.

**Figure 11:**
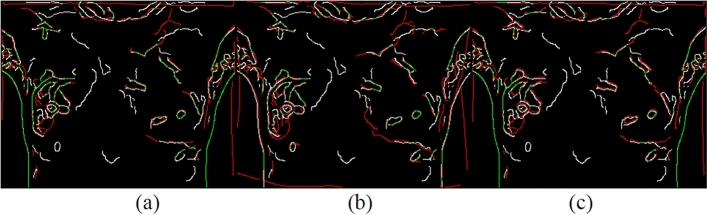
Canny overlays of TYPE III images obtained with (**a**) the proposed (**b**) Lee et al*.* (**c**) Optical flow.

**Table 4:** and Fig. 9, 10, 11, 12 demonstrate that the proposed method outperformed the method developed by Lee et al. in terms of both visualization and charting results. Therefore, the proposed method achieved better image registration for images with large displacements or many flat regions. Even in difficult cases, the algorithm proposed in this study performed well.

	Proposed(A)	Optical flow(B)	Lee et al*.* (C)	Significance statement (p-value)
Type I (15 cases)	1.141598 $\pm$ 0.0190168	1.132883 $\pm$ 0.0121690	1.131403 $\pm$ 0.0198858	(A,B) p-value = 0.001 (A,C) p-value = 0.000 (B,C) p-value = 0.585
Type II (8 cases)	1.147178 $\pm$ 0.0150833	1.138849 $\pm$ 0.0139354	1.139798 $\pm$ 0.0135367	(A,B) p-value = 0.001 (A,C) p-value = 0.001 (B,C) p-value = 0.001
Type III (6 cases)	1.132366 $\pm$ 0.0183055	1.124298 $\pm$ 0.0165760	1.122981 $\pm$ 0.0165402	(A,B) p-value = 0.025 (A,C) p-value = 0.000 (B,C) p-value = 0.615
Type IV (9 cases)	1.131028 $\pm$ 0.0278672	1.119034 $\pm$ 0.0254530	1.126921 $\pm$ 0.0261110	(A,B) p-value = 0.001 (A,C) p-value = 0.033 (B,C) p-value = 0.013

**Figure 12:**
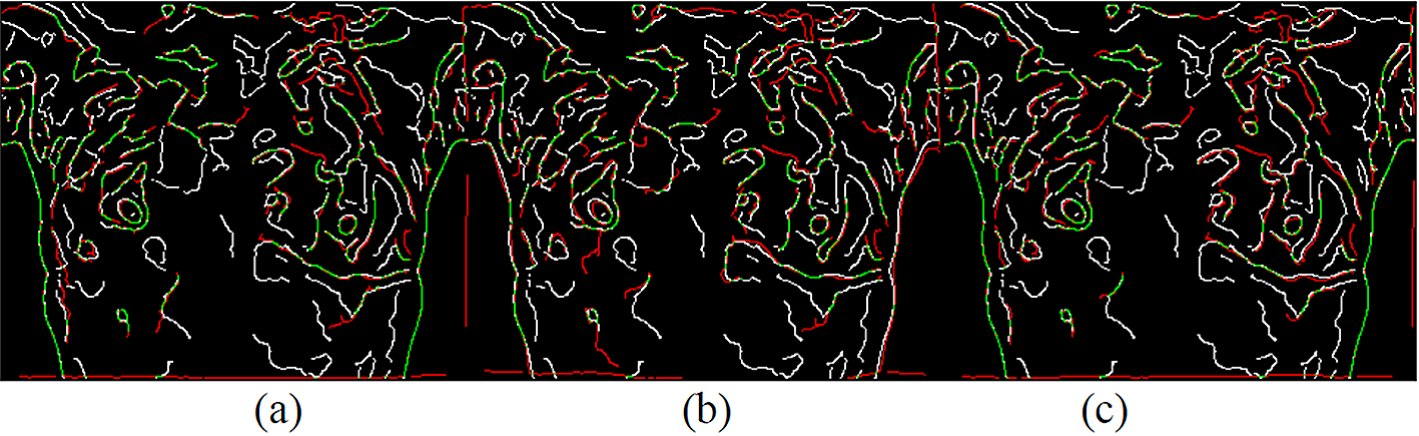
Canny overlays of TYPE IV images obtained with (**a**) the proposed (**b**) Lee et al*.* (**c**) Optical flow.

**Table 5:** Time performers of the proposed method in the four registration types.

(Unit: second)	Type I (15 cases)	Type II (8 cases)	Type III (6 cases)	Type IV (9 cases)
Proposed	23.744 $\pm$ 1.516	34.401 $\pm$ 3.691	27.346 $\pm$ 0.838	26.1806 $\pm$ 1.683
Optical flow	0.915 $\pm$ 0.094	0.899 $\pm$ 0.016	0.906 $\pm$ 0.037	0.885 $\pm$ 0.009
Lee et al	41.96 $\pm$ 3.814	42.847 $\pm$ 3.572	43.148 $\pm$ 2.906	42.989 $\pm$ 3.421

The difference analysis of the quantification results (MI) of the different methods are: TYPE I, TYPE III, and TYPE IV. Significant differences were observed between the proposed method and other methods (P < 0.05). For TYPE I and TYPE III images, the method developed by Lee et al*.* did not exhibit significantly different performance from that of LDOF, but a significant difference was observed between method developed by Lee et al*.* and the proposed method. These results demonstrate that the proposed method can outperform existing methods in terms of image registration in any registration situation.

According to the experimental results, although this method can achieve a good registration effect, it still has the following limitations. The method of this study is not effective for the case of surgically removed breasts, because it is difficult to find corresponding feature points in the images of the two time points for the surgically removed part. Although this research method is suitable for cases with large displacement, such as TYPE I and TYPE VI, if the patient's displacement in the images at two time points is too large, such as sideways, the registration cannot be achieved through this research method. Fortunately, patients are usually asked to maintain a similar posture during the filming of such images as they were in the previous filming. Due to the limitation of the feature point detection method, this research method is only suitable for registration of infrared breast images.

## Methods

**Figure 13:**
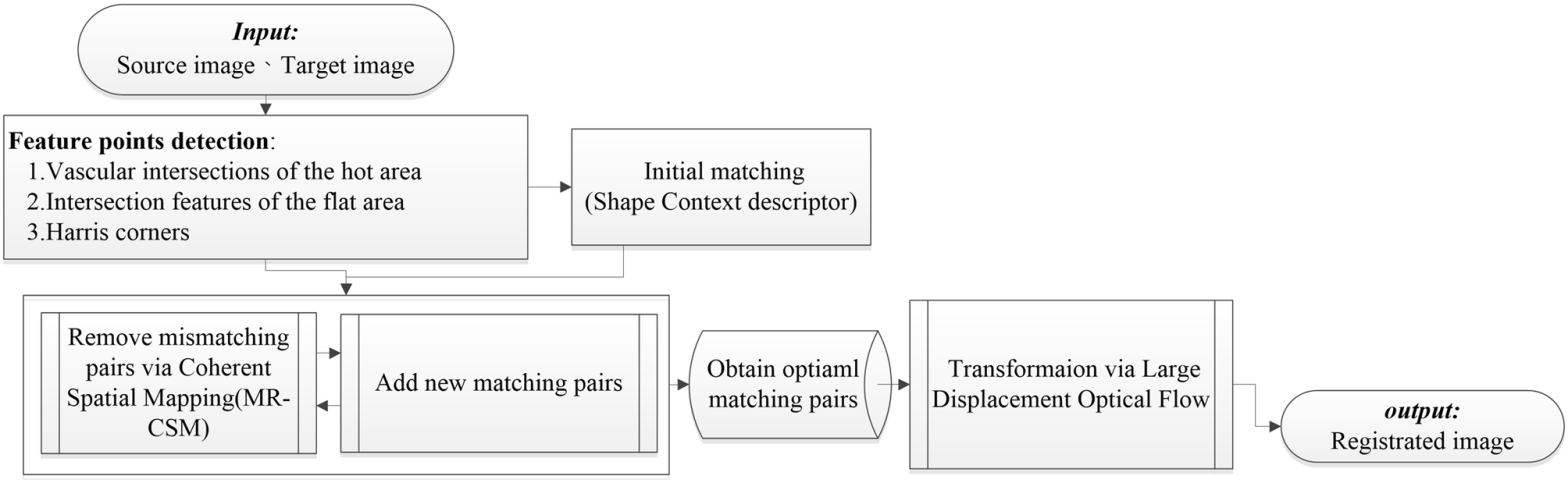
Flowchart of the proposed registration algorithm.

This study was approved by the institutional review board (IRB) of National Taiwan University Hospital (NTUH). Sixty-one (61) breast cancer patients who were between the ages of 25 and 72 (with an average age of 50) were recruited via the IRB-approved protocol from July 2011 to Jan 2013. The patients were treated using chemotherapy and examined using a quantitative dual-spectrum infrared system. The cancer treatment guidelines are strictly in accordance with the standards that have been set by the NTUH. All methods were performed in accordance with the relevant guidelines and regulations and informed consent was obtained from all subjects and/or their legal guardian(s). Two IR cameras (FLIR systems) were used to measure the 3–5-μm- and 8–9.2-μm-wavelength bands of IR radiation. The cancer detectors consist of 320*256 elements. The spatial resolution and temperature are approximately 0.6 mm and 0.02 degrees Celsius, respectively. Infrared breast feature point matching, in combination with the registration techniques that are discussed in this study, realizes maximum effectiveness and registration accuracy. Figure 13 presents a detailed explanation of the procedures.

In this study, a total of 38 multitemporal infrared images were divided into four registration situations according to the number of flat areas and the displacement degree of the target, as described in Sect. 2. The number of each type is shown in the Table 6. The multitemporal infrared images of each case consist of a set of source and target images taken at different time points.

**Table 6:** The number of four registration situations.

	Type I	Type II	Type III	Type VI	Total
Number of cases	15	8	6	9	38

### Feature point detection

**Figure 14:**
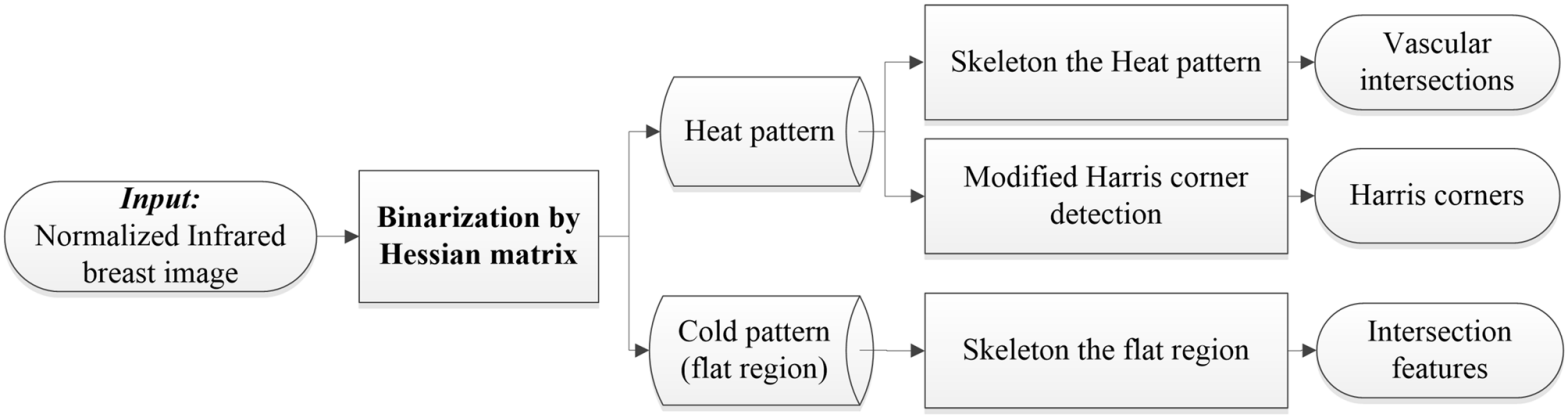
Flowchart of the feature detection process.

Three types of feature points are considered: vascular intersections and Harris corners in the heat pattern and intersection features in the cold pattern. A flow chart is shown in Fig. 14. The method of Harris corners and intersection features used in this study is from Lee et al.^28^. The process of each step is explained below, where the cold pattern corresponds to the flat region. In mathematics, a Hessian matrix is a block matrix that is composed of the second-order partial derivatives of a multivariate function. For an image, calculating the Hessian matrix eigenvalues of each pixel corresponds to calculating the principal curvature of each pixel. A positive principal curvature is referred to as convex and negative as concave. Therefore, this study uses the principal curvature to distinguish the heat pattern and the cold pattern of an infrared image.

**Figure 15:**
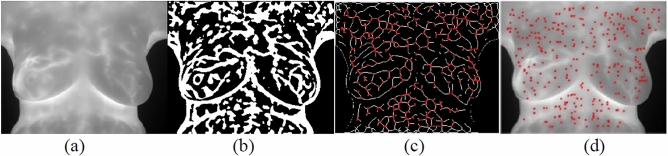
(**a**) An original image; (**b**) the binarization result; (**c**) the skeleton result of the cold pattern; and (**d**) the detection result of the cold pattern.

First, the image *I* is Gaussian-filtered into. Then, the Hessian matrix is established, as expressed in Eq. 1. Finally, the signs of eigenvalues with large absolute values are considered in (2). Figure 15 presents the result of feature point detection in a cold pattern.

Where ${\uplambda }_{1}$ and ${\uplambda }_{2}$ are the eigenvalues of the calculation matrix H. $\partial x$ and $\partial y$ represent the partial differentiation of the image *I* in the x and y directions, respectively. ${\mathrm{G}}_{\upsigma }$ is Gaussian-filter, where $\upsigma =2$.

This is the classic Frangi technique. By evaluating the value of the eigenvalue, it can be determined whether the calculation area is a bright area or a dark area. Here, the thermal imaging uses eigenvalue to distinguish it as a hot area and a cold area.

1 $$H={G}_{\sigma }*\left[\genfrac{}{}{0pt}{}{\frac{{\partial }^{2}I}{\partial {x}^{2}}}{\frac{{\partial }^{2}I}{\partial x\partial y}}\genfrac{}{}{0pt}{}{\frac{{\partial }^{2}I}{\partial x\partial y}}{\frac{{\partial }^{2}I}{\partial {y}^{2}}}\right]$$

2 $$Binarization\left\{\begin{array}{c}Hot pattren\left\{\begin{array}{c}{\lambda }_{2}<0,if\left|{\lambda }_{1}\right|<\left|{\lambda }_{2}\right|\\ {\lambda }_{1}<0,if\left|{\lambda }_{2}\right|<\left|{\lambda }_{1}\right|\end{array}\right.\\ Cold pattren\left\{\begin{array}{c}{\lambda }_{2}>0,if\left|{\lambda }_{1}\right|<\left|{\lambda }_{2}\right|\\ {\lambda }_{1}>0,if\left|{\lambda }_{2}\right|<\left|{\lambda }_{1}\right|\end{array}\right.\end{array}\right.$$

### Feature matching and optimization of image registration

After feature detection, Shape Context is used to establish a feature point descriptor, which is used to conduct initial matching via the proximity matrix (in which every distance between rows is minimal). The initially matched points are provided to the MR-CSM for optimization of the matching of the features, thereby reducing the constraint parameter sensitivity and the deficient local representability of matching to enhance the matching performance.

- Feature description

**Figure 16:**
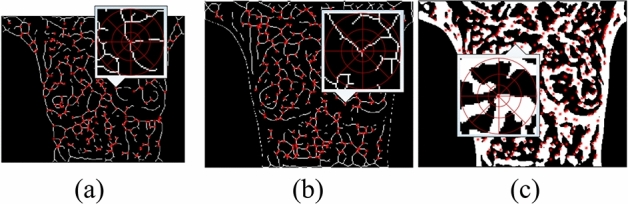
Shape context descriptors (**a**) for W points, (**b**) for B points, and (**c**) for C points. Feature point matching.

This study describes feature points in SC. For convenience, vascular intersections of the heat pattern, intersection features of the cold pattern, and Harris corners of the heat pattern are represented as W points, B points, and C points in the remainder of this paper. SC was conducted for W, B, and C points to obtain the descriptors, as shown in Fig. 16. The similarities between the descriptors of the target image and those of the source image are calculated as follows:

3 $${\mathrm{D}}_{\mathrm{w}}=\frac{1}{2}\sum_{\mathrm{k}=1}^{\mathrm{K}}\frac{{\left[{\mathrm{I}}_{2\mathrm{w}}\left(\mathrm{k}\right)-{\mathrm{I}}_{1\mathrm{w}}(\mathrm{k})\right]}^{2}}{{\mathrm{I}}_{2\mathrm{w}}\left(\mathrm{k}\right)+{\mathrm{I}}_{1\mathrm{w}}(\mathrm{k})}$$

4 $${\mathrm{D}}_{\mathrm{B}}=\frac{1}{2}\sum_{\mathrm{k}=1}^{\mathrm{K}}\frac{{\left[{\mathrm{I}}_{2\mathrm{B}}\left(\mathrm{k}\right)-{\mathrm{I}}_{1\mathrm{B}}(\mathrm{k})\right]}^{2}}{{\mathrm{I}}_{2\mathrm{B}}\left(\mathrm{k}\right)+{\mathrm{I}}_{1\mathrm{B}}(\mathrm{k})}$$

5 $${\mathrm{D}}_{C}=\frac{1}{2}\sum_{\mathrm{k}=1}^{\mathrm{K}}\frac{{\left[{\mathrm{I}}_{2\mathrm{C}}\left(\mathrm{k}\right)-{\mathrm{I}}_{1\mathrm{C}}(\mathrm{k})\right]}^{2}}{{\mathrm{I}}_{2\mathrm{C}}\left(\mathrm{k}\right)+{\mathrm{I}}_{1\mathrm{C}}(\mathrm{k})}$$

Among them, ${\mathrm{I}}_{2\mathrm{w}},{\mathrm{I}}_{2\mathrm{B}},{\mathrm{I}}_{2\mathrm{C}},{\mathrm{I}}_{1\mathrm{w}},{\mathrm{I}}_{1\mathrm{B}},{\mathrm{I}}_{1\mathrm{C}}$ are the SC descriptor of the above three feature points in the images at two time points respectively. k represents the order of each record square in the descriptor.

**Figure 17:**
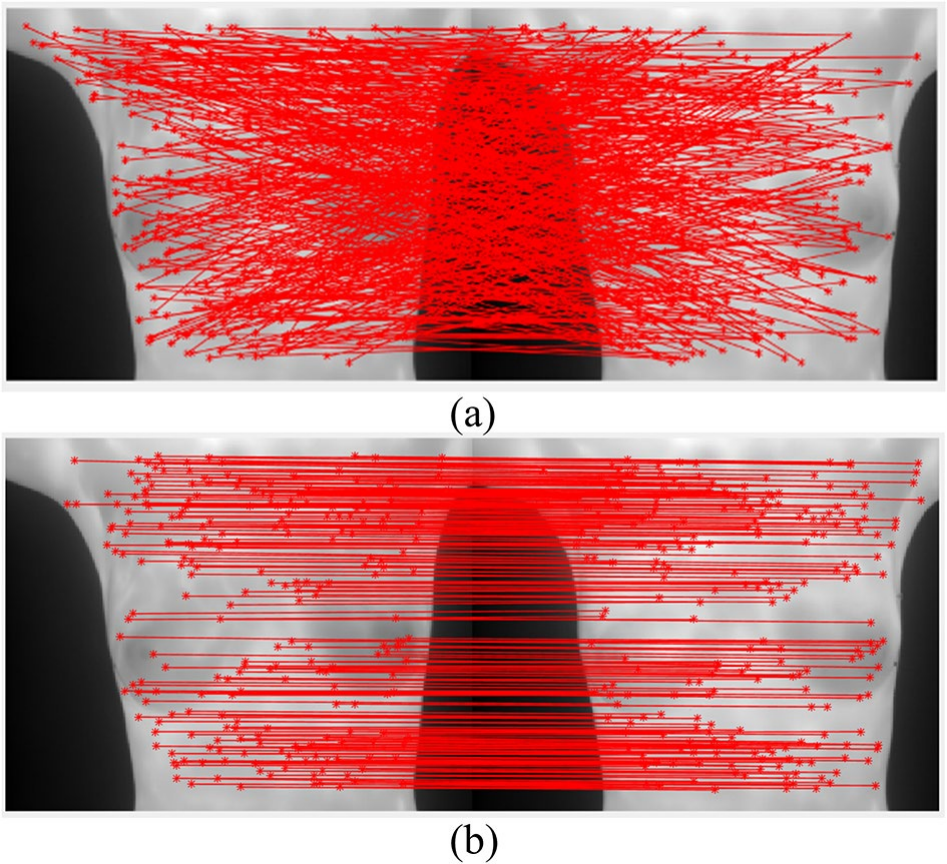
Matched points: (**a**) in the initial matching and (**b**) after deletion of the mismatched points.

When applying the SC to conduct the initial matching, the number of matching pairs remains insufficient and the pairs are unable to provide sufficient effective information for the transformation model. To overcome this problem, this study developed a matching algorithm that mutually iterates the two processes and effectively realizes the objective that is specified above. Initially, matching pairs are generated through matching with the SC descriptor. Then, stage one is entered, wherein the TPS model of the MR-CSM is used to remove mismatched points. The result is presented in Fig. 17, where the removed points are recorded. The pseudo-code of the matching in this study is shown in the Fig. 20a.

**Figure 18:**
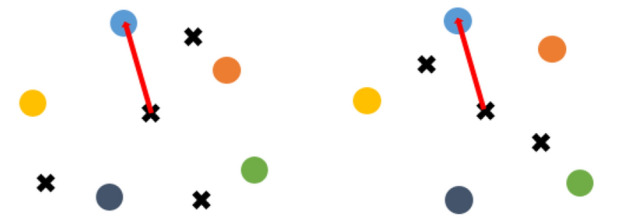
Illustration of the addition of the remaining matched points. Points of the same color are corresponding pairs.

Stage two: The matching pairs that were reserved in the first stage are used to add the remaining points. The relative distance that is used in the calculation is illustrated in Fig. 18, where circle points represent matching pairs from the first stage and cross points represent the remaining points to be added. Every pair that has similar relative distance from the matching pairs that remain after stage one is regarded as a candidate matching pair, and the calculation equation for the relative distance between matching pairs is presented in (6). Among them, ${P}_{1}$,${P}_{2}$ represent the coordinate of matching pairs of the target image and the source image, respectively. k represents the order of each record square in the descriptor. During the matching process in this study, the pseudo-code for adding correct matching pairs is shown in the Fig. 20b

6 $$\frac{1}{K}{\sum }_{k=1}^{K}\| {P}_{2k}-{P}_{1k}\| $$

**Figure 19:**
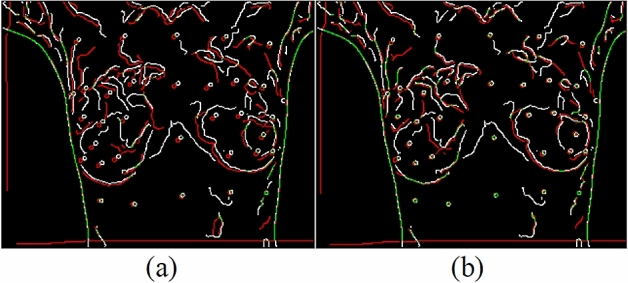
Registration results (**a**) without matching pairs and (**b**) with added matching pairs.

Among the candidate pairs, the pair with the minimum distance from the SC descriptor is selected as the matching pair. However, the pairs that were previously removed should be excluded. The above two steps are repeated until the matched points are stable. Figure 19 compares the results that are obtained with versus without stage two.

**Figure 20:**
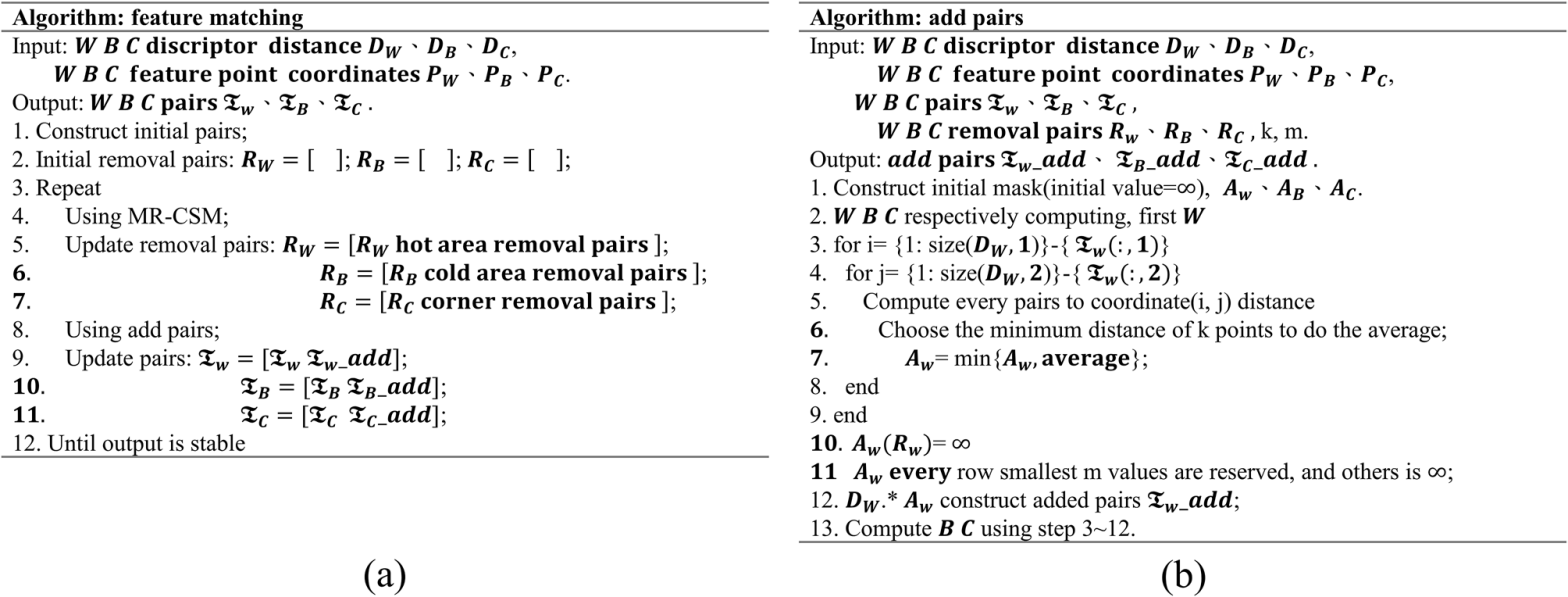
pseudo-code for (**a**) matching and (**b**) the addition of pairs.

### Image transformation: estimating the transform functions

**Figure 21:**
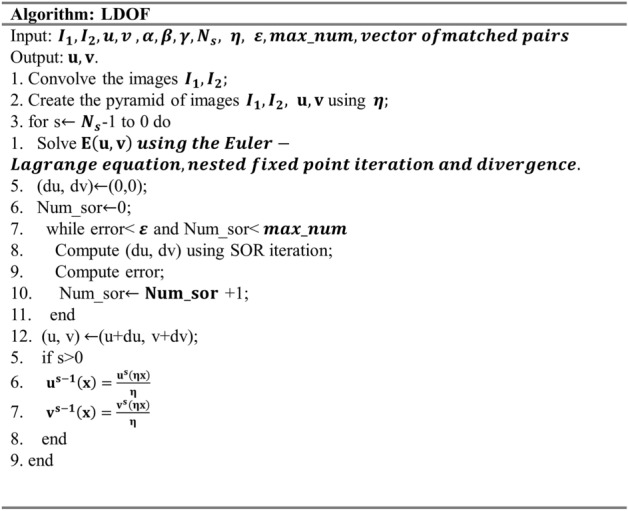
Pseudo-code of LDOF.

Upon completion of the matching, this study estimates the transform functions based on LDOF^41^. LDOF is an optical flow method that uses matched point pairs to realize a "large displacement" and adds gradient information to increase the tolerance to grayscale changes in images. LDOF obtains the displacement $\mathrm{u},\mathrm{v}$ by solving the energy function E(w) by inputting the two task images and vector of the matching pairs. Its pseudo code is shown in the Fig. 21.

The energy function E is composed of four terms shows in (13), where $\mathbf{w}={\left(\mathrm{u},\mathrm{v}\right)}^{\mathrm{T}}$ is the sought displacement field, and x: = (x, y) denotes a point in the image. α, β, and γ are tuning parameters representing the importance of smoothness, region correspondences, and gradient stability respectively^41^. In this research we set γ = 20, α = 50 and β = 10,000. The value of β must be set a larger, because this study focuses on the accuracy of matching, followed by the smoothness of deformation. Because of the distribution of gradient for IR breast image registration is not the main consideration therefore the parameter γ is smaller. The details of solving this energy function in this study can be found in Attachment 1

$$\mathrm{E}\left(\mathbf{w}\left(\mathbf{x}\right)\right)={\mathrm{E}}_{\mathrm{color}}\left(\mathbf{w}\left(\mathbf{x}\right)\right)+\upgamma {\mathrm{E}}_{\mathrm{gradient}}\left(\mathbf{w}\left(\mathbf{x}\right)\right)$$

13 $$+\mathrm{\alpha }{\mathrm{E}}_{\mathrm{smooth}}\left(\mathbf{w}(\mathbf{x})\right)+\upbeta {\mathrm{E}}_{\mathrm{match}}\left(\mathbf{w}(\mathbf{x})\right)$$

Solving the energy function $\mathrm{E}\left(\mathbf{w}\right)$ using the Euler–Lagrange equation, nested fixed point iteration and divergence. Finally, successive over-relaxation (SOR) iteration is used to obtain $\mathrm{du}$ and $\mathrm{dv}$. Add the current displacement field w to the calculated $\mathrm{d}\mathbf{w}$ to obtain the updated displacement field as shown in Eq. (14), where k represents the current number of updates. The iteration termination condition of SOR iteration is to calculate the error defined by Eq. (15)

14 $${{\mathrm{d}\mathbf{w}}^{\mathrm{k}+1}=\mathbf{w}+\mathrm{d}\mathbf{w}}^{\mathrm{k}}$$

15 $$\mathrm{error}=\frac{1}{\mathrm{N}}\sum_{\mathrm{i},\mathrm{j}}\left({\left({\mathrm{du}}^{\mathrm{k}+1}-{\mathrm{du}}^{\mathrm{k}}\right)}^{2}+{\left({\mathrm{dv}}^{\mathrm{k}+1}-{\mathrm{dv}}^{\mathrm{k}}\right)}^{2}\right)<\upvarepsilon $$

where $\mathrm{N}$ is the total number of combinations i, j. $\upvarepsilon$ is the settable threshold of error and k is the number of SOR iteration.The coarse-to-fine process is used to estimate the large displacement field. In the process, a pyramid image must be established, where Gaussian filtering is used during downsampling to make the process smoother. Then, the process starts from the large scale (coarse) and slowly updates to the small scale (fine) as Eqs. (16), (17), where s is the scale which is changed by scale factor η ∈ (0, 1).

16 $${\mathrm{u}}^{\mathrm{s}-1}\left(\mathbf{x}\right)=\frac{{\mathrm{u}}^{\mathrm{s}}\left(\upeta \mathbf{x}\right)}{\upeta }$$

17 $${\mathrm{v}}^{\mathrm{s}-1}\left(\mathbf{x}\right)=\frac{{\mathrm{v}}^{\mathrm{s}}\left(\upeta \mathbf{x}\right)}{\upeta }$$

## Conclusions

Automated infrared breast imaging registration via quantitative analysis of the completed registration of heat pattern information provides an efficient, noninvasive, and non-radioactive method for tissue-growth assessment and early tumor detection in breast cancers. This study consists of three major parts and proposes a novel infrared image registration algorithm. In the first part, via a Hessian matrix calculation, this method obtains representative grid intersections of a flat region, and the feature points that are obtained in combination with the algorithm by Lee et al. This study also verified that the feature point detection method proposed in this study meets the requirements of sufficient, uniformly distributed, and representative feature points through the results of visualization and quantitative analysis in the four registration conditions. In the second part, based on the MR-CSM algorithm, a two-stage alternating iterative method was developed for correcting erroneous matching results and continuously recording new matched points to realize optimal feature point matching. In the matching verification, the four registration situations are also used to verify that the proposed method has better matching performance than other classic methods in terms of matching precision and matching sparsity. In the third part, the matched point information that as obtained via the feature point matching strategy is provided to the LDOF algorithm for estimation of the transform functions, and using the concept of pixel-level displacement, this method conducts LDOF deformation to obtain an optimized image registration. In the results that are obtained by the improved algorithm, the Canny overlay edges in the visualization values demonstrate effective registration. Comparing the results obtained by the improved algorithm proposed in this research with the results of other different matching descriptors and transformation models, it can be concluded that the method proposed in this research obtains the best registration performance. It has effective registration performance in the visual verification of Canny coverage edge in the four registration situations. The registration algorithm that is proposed in this study also promotes the development of medical imaging information and enhances the potential benefits of infrared imaging for breast cancer diagnosis. The future work of this research includes two aspects. In terms of method, this research is expected to collect more data to further verify the robustness of this research, and apply it to the development of deep learning models based on the proposed methods. In terms of application, this study is expected to use this registration tool to register the infrared breast images of before and after chemotherapy, and then obtain non-invasive quantitative indicators of chemotherapy effects through thermal map analysis of the corresponding affected area.

## Supplementary Information

Supplementary Information.
