# Supplementary material for: Image registration method using representative feature detection and iterative coherent spatial mapping for infrared medical images with flat regions
Source: Sci Rep. 2022 May 13;12:7932. doi: 10.1038/s41598-022-11379-2 (PMC9106756; doi:10.1038/s41598-022-11379-2)
Supplement: Supplementary file 1 — Supplementary Information. [file 41598_2022_11379_MOESM1_ESM.docx]

LDOF is an optical flow method that uses matched point pairs to realize a "large displacement" and adds gradient information to increase the tolerance to grayscale changes in images [41]. It obtains the displacement $u,v$ by solving the energy function $E\left( \mathbf{w} \right)$ by inputting the two task images and vector of the matching pairs. The energy function E is composed of four terms shows in Eq. (1)-(5), where $\mathbf{w}:=(u,v)$ is the sought displacement field, and $\mathbf{x}$: = (x, y) denotes a feature point in the image $I_{1}\mathrm{and} I_{2}$. α, β, and γ are tuning parameters representing the importance of smoothness, region correspondences, and gradient stability, respectively [41]. In this research we set γ = 20、α= 50 and β= 10000. The value of β must be set a larger, because this study focuses on the accuracy of matching, followed by the smoothness of deformation. Because of the distribution of gradient for IR breast image registration is not the main consideration therefore the parameter γ is smaller.

$E\left( \mathbf{w} \right)=E_{\mathrm{color}}\left( \mathbf{w} \right)+\gamma E_{\mathrm{gradient}}\left( \mathbf{w} \right)+\alpha E_{\mathrm{smooth}}\left( \mathbf{w} \right)+\beta E_{\mathrm{match}}\left( \mathbf{w} \right)$ (1)

The matching pair’s displacement $\mathbf{w}_{1}$ is expressed in (6); the matching score ρ is expressed in (7), in which nor denotes normalization to 0–1; the pulse δ(x) is expressed in (8). It is 1 at the position of the matching point pair; and $\mathbf{x}_{1}$ and $\mathbf{x}_{2}$ are the positions of matched points in the target and source images, respectively.

$E_{\mathrm{color}}\left( \mathbf{w} \right)=\int_{\Omega} \Psi\left( \left| I_{2}\left( \mathbf{x}+\mathbf{w}\left( \mathbf{x} \right) \right)-I_{1}\left( \mathbf{x} \right) \right|^{2} \right)d\mathbf{x}$ (2)

$E_{\mathrm{gradient}}\left( \mathbf{w} \right)=\int_{\Omega} \Psi\left( \left| \nabla I_{2}\left( \mathbf{x}+\mathbf{w}\left( \mathbf{x} \right) \right)-\nabla I_{1}\left( \mathbf{x} \right) \right|^{2} \right)d\mathbf{x}$ (3)

$E_{\mathrm{smooth}}\left( \mathbf{w} \right)=\int_{\Omega} \Psi\left( \left| \nabla u\left( \mathbf{x} \right) \right|^{2}+\left| \nabla v\left( \mathbf{x} \right) \right|^{2} \right)d\mathbf{x}$ (4)

$E_{\mathrm{match}}\left( \mathbf{w} \right)=\int_{\Omega} \delta\left( \mathbf{x} \right)\rho\left( \mathbf{x} \right)\Psi\left( \left| \mathbf{w}\left( \mathbf{x} \right)-\mathbf{w}_{1}\left( \mathbf{x} \right) \right|^{2} \right)d\mathbf{x}$ (5)

$\mathbf{w}_{1}\left( \mathbf{x} \right)=\mathbf{x}_{2}\left( \mathbf{x} \right)-\mathbf{x}$ (6)

$\rho=nor(\sqrt{(1-{nor(D}_{\left\{ W,B,C \right\}}))(1-nor(\left\| \mathbf{x}_{2}-\mathbf{x}_{1} \right\|))})$ (7)

$\delta\left( \mathbf{x} \right)\left\{ \begin{aligned} 1,\mathbf{x=}\mathbf{x}_{1} \\ 0,\boldsymbol{x\neq}\mathbf{x}_{1} \end{aligned} \right.$ (8)

where $\Psi\left( s \right)=\sqrt{s+\epsilon}; \mathbf{x}$ is the position and $\mathbf{x}:=\left( x,y \right)^{T}; \rho$denotes the paired points for matching$;\mathrm{and} \mathbf{w}_{1}$ is the vector of the matching point pairs. Solving E using the Euler-Lagrange equation yields the following formula:

$\Psi^{'}\left( I_{z}^{2} \right)I_{z}I_{x}+\gamma\Psi^{'}\left( I_{\mathrm{xz}}^{2}+I_{\mathrm{yz}}^{2} \right)\left( I_{\mathrm{xx}}I_{\mathrm{xz}}+I_{\mathrm{xy}}I_{\mathrm{yz}} \right)+\beta\rho\Psi^{'}\left( \left( u-u_{1} \right)^{2}+\left( v-v_{1} \right)^{2} \right)\left( u-u_{1} \right)-\alpha div\left( \Psi^{'}\left( \left| \nabla u \right|^{2}+\left| \nabla v \right|^{2} \right)\nabla u \right)=0$ (9)

$\Psi^{'}\left( I_{z}^{2} \right)I_{z}I_{y}+\gamma\Psi^{'}\left( I_{\mathrm{xz}}^{2}+I_{\mathrm{yz}}^{2} \right)\left( I_{\mathrm{xy}}I_{\mathrm{xz}}+I_{\mathrm{yy}}I_{\mathrm{yz}} \right)+\beta\rho\Psi^{'}\left( \left( u-u_{1} \right)^{2}+\left( v-v_{1} \right)^{2} \right)\left( v-v_{1} \right)-\alpha div\left( \Psi^{'}\left( \left| \nabla u \right|^{2}+\left| \nabla v \right|^{2} \right)\nabla v \right)=0$ (10)

$I_{x}:=\partial_{x}I_{2}(\mathbf{x}+\mathbf{w})$ (11)

$I_{y}:=\partial_{y}I_{2}(\mathbf{x}+\mathbf{w})$ (12)

$I_{z}:=I_{2}\left( \mathbf{x}+\mathbf{w} \right)-I_{1}(\mathbf{x})$ (13)

$I_{\mathrm{xx}}:=\partial_{\mathrm{xx}}I_{2}(\mathbf{x}+\mathbf{w})$ (14)

$I_{\mathrm{xy}}:=\partial_{\mathrm{xy}}I_{2}(\mathbf{x}+\mathbf{w})$ (15)

$I_{\mathrm{yy}}:=\partial_{\mathrm{yy}}I_{2}(\mathbf{x}+\mathbf{w})$ (16)

$I_{\mathrm{xz}}:=\partial_{x}I_{z}$ (17)

$I_{\mathrm{yz}}:=\partial_{y}I_{z}$ (18)

Nested fixed point iteration is used to overcome the difficulty of solving $I_{2}\left( \mathbf{x}+\mathbf{w} \right)$ with a non-linear function, and the above formula can be re-expressed as:

$0=\Psi_{D}^{'}I_{x}^{k}\left( I_{\mathcal{z}}^{k}+I_{x}^{k}du^{k}+I_{y}^{k}dv^{k} \right)+\beta\rho\Psi_{M}^{'}\left( u^{k}+du^{k}-u_{1} \right)+\gamma\Psi_{G}^{'}I_{\mathrm{xx}}^{k}\left( I_{\mathrm{xz}}^{k}+I_{\mathrm{xx}}^{k}du^{k}+I_{\mathrm{xy}}^{k}dv^{k} \right)+\gamma\Psi_{G}^{'}I_{\mathrm{xy}}^{k}\left( I_{\mathrm{yz}}^{k}+I_{\mathcal{x}y}^{k}du^{k}+I_{\mathrm{yy}}^{k}dv^{k} \right)-\alpha div\left( \Psi_{S}^{'}\nabla(u^{k}+\mathrm{du}^{k}) \right)$ (19)

$0=\Psi_{D}^{'}I_{x}^{k}\left( I_{\mathcal{z}}^{k}+I_{x}^{k}du^{k}+I_{\mathcal{y}}^{k}dv^{k} \right)+\beta\rho\Psi_{M}^{'}\left( v^{k}+dv^{k}-v_{1} \right)+\gamma\Psi_{G}^{'}I_{\mathrm{xy}}^{k}\left( I_{\mathcal{x}z}^{k}+I_{\mathrm{xx}}^{k}du^{k}+I_{\mathrm{xy}}^{k}dv^{k} \right)+\gamma\Psi_{G}^{'}I_{\mathrm{yy}}^{k}\left( I_{\mathrm{yz}}^{k}+I_{\mathrm{xy}}^{k}du^{k}+I_{\mathrm{yy}}^{k}dv^{k} \right)-\alpha div\left( \Psi_{S}^{'}\nabla(v^{k}+dv^{k}) \right)$ (20)

$\Psi_{D}^{'}:=\Psi^{'}\left( \left( I_{z}^{k}+I_{x}^{k}du^{k}+I_{y}^{k}dv^{k} \right)^{2} \right)$ (21)

$\Psi_{G}^{'}:=\Psi^{'}\left( \left( I_{\mathrm{xz}}^{k}+I_{\mathrm{xx}}^{k}du^{k}+I_{\mathrm{xy}}^{k}dv^{k} \right)^{2}+\left( I_{\mathrm{yz}}^{k}+I_{\mathrm{xy}}^{k}du^{k}+I_{\mathrm{yy}}^{k}dv^{k} \right)^{2} \right)$ (22)

$\Psi_{M}^{'}:=\Psi^{'}\left( \left( u^{k}+du^{k}-u_{1} \right)^{2}+\left( v^{k}+dv^{k}-v_{1} \right)^{2} \right)$ (23)

$\Psi_{S}^{'}:=\Psi^{'}\left( {|\nabla\left( u^{k}+du^{k} \right)|}^{2}+{|\nabla\left( v^{k}+dv^{k} \right)|}^{2} \right)$ (24)

The divergence in the differentiation process can be represented as a difference as follows:

$\mathrm{div}\left( \Psi_{S}^{'}\nabla\left( u^{k}+\mathrm{du}^{k} \right) \right)=div\left( \Psi_{S}^{'}\nabla u^{k} \right)+div\left( \Psi_{S}^{'}\nabla\mathrm{du}^{k} \right)\approx divu+\left( divdu-divd\cdot du \right)$ (25)

$divu=\frac{\Psi_{S}^{'}\left( i+1,j \right)+\Psi_{S}^{'}\left( i,j \right)}{2}\left( u\left( i+1,j \right)+u\left( i,j \right) \right)+\frac{\Psi_{S}^{'}\left( i-1,j \right)+\Psi_{S}^{'}\left( i,j \right)}{2}\left( u\left( i-1,j \right)+u\left( i,j \right) \right)+\frac{\Psi_{S}^{'}\left( i,j+1 \right)+\Psi_{S}^{'}\left( i,j \right)}{2}\left( u\left( i,j+1 \right)+u\left( i,j \right) \right)+\frac{\Psi_{S}^{'}\left( i,j-1 \right)+\Psi_{S}^{'}\left( i,j \right)}{2}\left( u\left( i,j-1 \right)+u\left( i,j \right) \right)$ (26)

$divdu=\frac{\Psi_{S}^{'}\left( i+1,j \right)+\Psi_{S}^{'}\left( i,j \right)}{2}\mathrm{du}\left( i+1,j \right)+\frac{\Psi_{S}^{'}\left( i-1,j \right)+\Psi_{S}^{'}\left( i,j \right)}{2}\mathrm{du}\left( i-1,j \right)+\frac{\Psi_{S}^{'}\left( i,j+1 \right)+\Psi_{S}^{'}\left( i,j \right)}{2}\mathrm{du}\left( i,j+1 \right)+\frac{\Psi_{S}^{'}\left( i,j-1 \right)+\Psi_{S}^{'}\left( i,j \right)}{2}\mathrm{du}\left( i,j-1 \right)$ (27)

$divd=\frac{\Psi_{S}^{'}\left( i+1,j \right)+\Psi_{S}^{'}\left( i,j \right)}{2}+\frac{\Psi_{S}^{'}\left( i-1,j \right)+\Psi_{S}^{'}\left( i,j \right)}{2}+\frac{\Psi_{S}^{'}\left( i,j+1 \right)+\Psi_{S}^{'}\left( i,j \right)}{2}+\frac{\Psi_{S}^{'}\left( i,j-1 \right)+\Psi_{S}^{'}\left( i,j \right)}{2}$ (28)

Finally, SOR iteration is used to obtain $\mathrm{du}$ and $\mathrm{dv}$ as follows:

$du^{k+1}=\left( 1-\omega\right)du^{k}+\omega\frac{Au-D\cdot dv^{k}+\alpha divdu}{\mathrm{Du}}$ (29)

$dv^{k+1}=\left( 1-\omega\right)dv^{k}+\omega\frac{Av-D\cdot du^{k}+\alpha divdv}{\mathrm{Dv}}$ (30)

$Au=-\Psi_{D}^{'}I_{z}I_{x}+\alpha divu-\gamma\Psi_{G}^{'}\left( I_{\mathrm{xz}}I_{\mathrm{xx}}+I_{\mathrm{yz}}I_{\mathrm{xy}} \right)-\beta\rho\Psi_{M}^{'}\left( u-u_{1} \right)$ (31)

$Av=-\Psi_{D}^{'}I_{z}I_{y}+\alpha divv-\gamma\Psi_{G}^{'}\left( I_{\mathrm{xz}}I_{\mathrm{xy}}+I_{\mathrm{yz}}I_{\mathrm{yy}} \right)-\beta\rho\Psi_{M}^{'}\left( v-v_{1} \right)$ (32)

$Du=\Psi_{D}^{'}{I_{x}}^{2}+\alpha divd-\gamma\Psi_{G}^{'}\left( {I_{\mathrm{xx}}}^{2}+{I_{\mathrm{xy}}}^{2} \right)+\beta\rho\Psi_{M}^{'}$ (33)

$Dv=\Psi_{D}^{'}{I_{y}}^{2}+\alpha divd-\gamma\Psi_{G}^{'}\left( {I_{\mathrm{yy}}}^{2}+{I_{\mathrm{xy}}}^{2} \right)+\beta\rho\Psi_{M}^{'}$ (34)

$D=\Psi_{D}^{'}I_{x}I_{y}+\gamma\Psi_{G}^{'}\left( I_{\mathrm{xx}}+I_{\mathrm{yy}} \right)I_{\mathrm{xy}}$ (35)

Add the current displacement field w to the calculated $d\mathbf{w}$ to obtain the updated displacement field as shown in Eq. (36), where k represents the current number of updates. The iteration termination condition can be defined by the Eq. (37).

${{d\mathbf{w}}^{k+1}=\mathbf{w}+d\mathbf{w}}^{k}$ (36)

$error=\frac{1}{N}\sum_{i,j} \left( \left( du^{k+1}-du^{k} \right)^{2}+\left( dv^{k+1}-dv^{k} \right)^{2} \right)<\varepsilon$ (37)

The coarse-to-fine process is used to estimate the large displacement field. Since the effect of δ on the fine scale is small, the effect of δ is magnified by the coarse scale. In the coarse-to-fine process, a pyramid image must be established, where Gaussian filtering is used during down sampling to make the process smoother. Then, the process starts from the coarse scale and slowly updates to the fine scale as Eq. (38)、Eq. (39), according to the scale factor η∈ (0, 1).

$u^{s-1}\left( \mathbf{x} \right)=\frac{u^{s}\left( \eta\mathbf{x} \right)}{\eta}$ (38)

$v^{s-1}\left( \mathbf{x} \right)=\frac{v^{s}\left( \eta\mathbf{x} \right)}{\eta}$ (39)
